# Luminescent Cyclometalated Iridium(III) Polypyridine Complexes Modified with a Dendritic Guanidinium Moiety as Molecular Glues for Combined Chemo-Photodynamic Therapy

**DOI:** 10.1021/acs.inorgchem.5c01970

**Published:** 2025-07-17

**Authors:** Jia-Hao Wang, Lawrence Cho-Cheung Lee, Alex Man-Hei Yip, Peter Kam-Keung Leung, Kou Okuro, Kenneth Kam-Wing Lo

**Affiliations:** † Department of Chemistry, 53025 City University of Hong Kong, Tat Chee Avenue, Kowloon, Hong Kong; ‡ Laboratory for Synthetic Chemistry and Chemical Biology Limited, Units 1503−1511, 15/F, Building 17 W, Hong Kong Science Park, Pak Shek Kok, New Territories, Hong Kong; § State Key Laboratory of Terahertz and Millimeter Waves, 53025 City University of Hong Kong, Tat Chee Avenue, Kowloon, Hong Kong; ∥ State Key Laboratory of Synthetic Chemistry and Department of Chemistry, The University of Hong Kong, Pokfulam Road, Pokfulam, Hong Kong

## Abstract

We report herein three cyclometalated iridium­(III) polypyridine complexes appended with a dendritic guanidinium unit [Ir­(N^C)_2_(bpy-Gu_9_)]­(Cl)_10_ (bpy-Gu_9_ = 4-(*N*-(3,4,5-tris­(2-(2-(2-(4-((3,4,5-tris­(2-(2-(2-guanidinoethoxy)­ethoxy)­ethoxy)­benz-amido)­methyl)-1*H*-1,2,3-triazol-1-yl)­ethoxy)­ethoxy)­ethoxy)­phenylcarbonyl)­aminomethyl)-4’-methyl-2,2’-bipyridine; HN^C = 2-phenylpyridine (Hppy) (**1a**), 2-phenylquinoline (Hpq) (**2a**), and 2-(1-naphthyl)­benzothiazole (Hbsn) (**3a**)) as molecular glues. Their guanidinium-free counterparts [Ir­(N^C)_2_(bpy-C4)]­(Cl) (bpy-C4 = 4-(*N*-(*n*-propylcarbonyl)­aminomethyl)-4’-methyl-2,2’-bipyridine; HN^C = Hppy (**1b**), Hpq (**2b**), and Hbsn (**3b**)) were also isolated. Irradiation of the complexes led to intense greenish-yellow to red emission. Additionally, the cellular uptake and (photo)­cytotoxic effects of the complexes were investigated. Protein-binding studies showed that the decacationic complexes **1a**–**3a** exhibited high binding affinity toward bovine serum albumin (BSA). Complex **1a** was utilized to modify doxorubicin (DOX)-loaded, glutathione (GSH)-responsive BSA nanoparticles (DOX/^SS^BNPs), affording Ir-DOX/^SS^BNPs. Notably, functionalization of DOX/^SS^BNPs with the complex reversed the surface charge from negative to positive. The size of Ir-DOX/^SS^BNPs decreased significantly upon incubation with GSH as a result of efficient DOX release. The positively charged Ir-DOX/^SS^BNPs showed efficient cellular uptake in HeLa cells, which proved to be crucial for their effectiveness in combined chemo-photodynamic therapy. These results showed that iridium­(III)-based molecular glues can be employed for drug delivery and combined chemo-photodynamic therapy.

## Graphical abstract

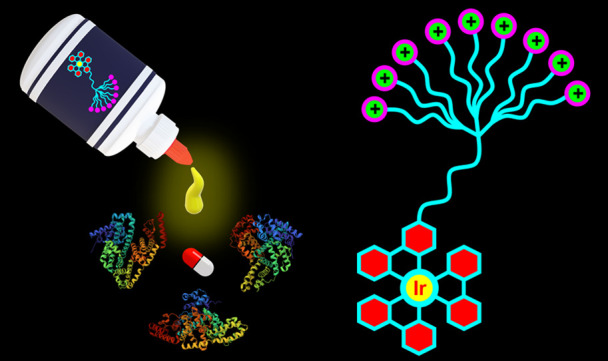

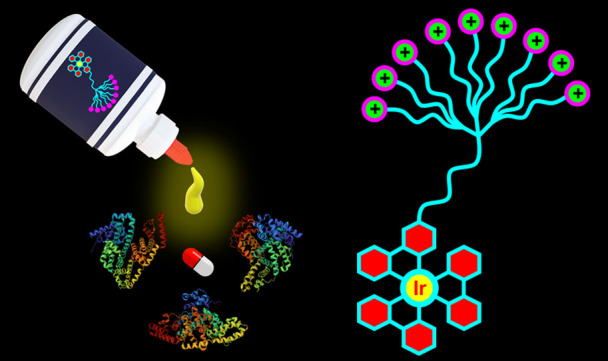

## Introduction

In living systems, noncovalent interactions are essential for facilitating biological processes by enabling specific binding between biomolecules.^1,2^ These interactions, while individually weak, can become significantly stronger when multiple interactions occur simultaneously, resulting in effective adhesion. For instance, antibodies bind to antigens on pathogen surfaces through multiple sites, enhancing immune recognition and clearance.^3^ This multivalent binding effectively inhibits infections and promotes the removal of foreign particles by macrophages. Another example of polyvalent noncovalent interactions is the retinoid X receptor (RXR) in gene regulation.^4^ The RXR–ligand complexes bind to DNA with increasing affinity as oligomerization occurs. This sensitivity to RXR concentration enhances transcriptional response, demonstrating the significance of polyvalent interactions in biological processes. Thus, noncovalent interactions allow biomolecules to function accurately and effectively in living systems, and regulation of intermolecular noncovalent interactions represents a powerful therapeutic strategy.

Molecular glues are a class of small molecules that can adhere to biomolecules and modulate their functions, demonstrating high therapeutic potential.^5^ Natural products such as tacrolimus and rapamycin have been approved by the FDA as molecular glues due to their capabilities to inhibit protein–protein interaction by physically blocking the recognition site of the target protein.^6^ Another example is paclitaxel (Taxol), which stabilizes tubulin heterodimer conformation, disrupting microtubule dynamics and exhibiting significant antitumor activity.^7,8^ These natural products showcase the therapeutic potential of molecular glues. Based on multiple noncovalent interactions, Kinbara, Aida, and co-workers first reported the development of guanidinium-based molecular glues with excellent water solubility and biocompatibility.^9^ These molecular glues contain multiple guanidinium pendants, facilitating the formation of multivalent salt bridges with negatively charged functional groups on target proteins,^9–16^ nucleic acids,^17–19^ and phospholipids.^20^ These compounds have been widely explored as an adhesion moiety for regulating biomolecular activity^13,14^ and for drug delivery applications.^16,17,19^

Photofunctional transition metal complexes are increasingly being recognized for their potential in bioimaging^21–23^ and phototherapeutic^24–26^ applications. We anticipate that the integration of a dendritic guanidinium unit into luminescent transition metal complexes will provide them with enhanced water solubility, better biocompatibility, and noncovalent binding capability. This will allow them to serve as photofunctional molecular glues for various biological and biomedical applications. Cyclometalated iridium­(ΙΙΙ) polypyridine complexes are especially intriguing because of their diverse structures and flexible synthesis options, along with their interesting photophysics and photochemistry, and adjustable cellular uptake and localization behavior.^27–29^ Although hydrophobic luminescent iridium­(ΙΙΙ) complexes containing substrates such as biotin,^30–33^ indole,^34^ and estrogen^35^ have been developed and utilized for noncovalent protein binding, water-soluble complexes bearing a dendritic guanidinium moiety have not been explored. Herein, we report three cyclometalated iridium­(III) polypyridine complexes appended with a dendritic guanidinium unit [Ir­(N^C)_2_(bpy-Gu_9_)]­(Cl)_10_ (bpy-Gu_9_ = 4-(*N*-(3,4,5-tris­(2-(2-(2-(4-((3,4,5-tris­(2-(2-(2-guanidinoethoxy)­ethoxy)­ethoxy)­benzamido)­methyl)-1*H*-1,2,3-triazol-1-yl)­ethoxy)­ethoxy)­ethoxy)­phenylcarbonyl)­aminomethyl)-4’-methyl-2,2’-bipyridine; HN^C = 2-phenylpyridine (Hppy) (**1a**), 2-phenylquinoline (Hpq) (**2a**), and 2-(1-naphthyl)­benzothiazole (Hbsn) (**3a**)) (Chart 1) as molecular glues. Their guanidinium-free counterparts [Ir­(N^C)_2_(bpy-C4)]­(Cl) (bpy-C4 = 4-(*N*-(*n*-propylcarbonyl)­aminomethyl)-4’-methyl-2,2’-bipyridine; HN^C = Hppy (**1b**), Hpq (**2b**), and Hbsn (**3b**)) were also isolated. The photophysical and singlet oxygen (^1^O_2_) photosensitization properties, cellular uptake, intracellular distribution, and (photo)­cytotoxic effects of the complexes were examined. The adhesion of the guanidinium complexes **1a**–**3a** to bovine serum albumin (BSA) was also studied. Furthermore, guanidinium complex-modified, doxorubicin (DOX)-loaded, glutathione (GSH)-responsive BSA nanoparticles (Ir-DOX/^SS^BNPs) were constructed. The applications of Ir-DOX/^SS^BNPs in intracellular DOX delivery and combined chemo-photodynamic therapy were also examined.

**Chart 1:**
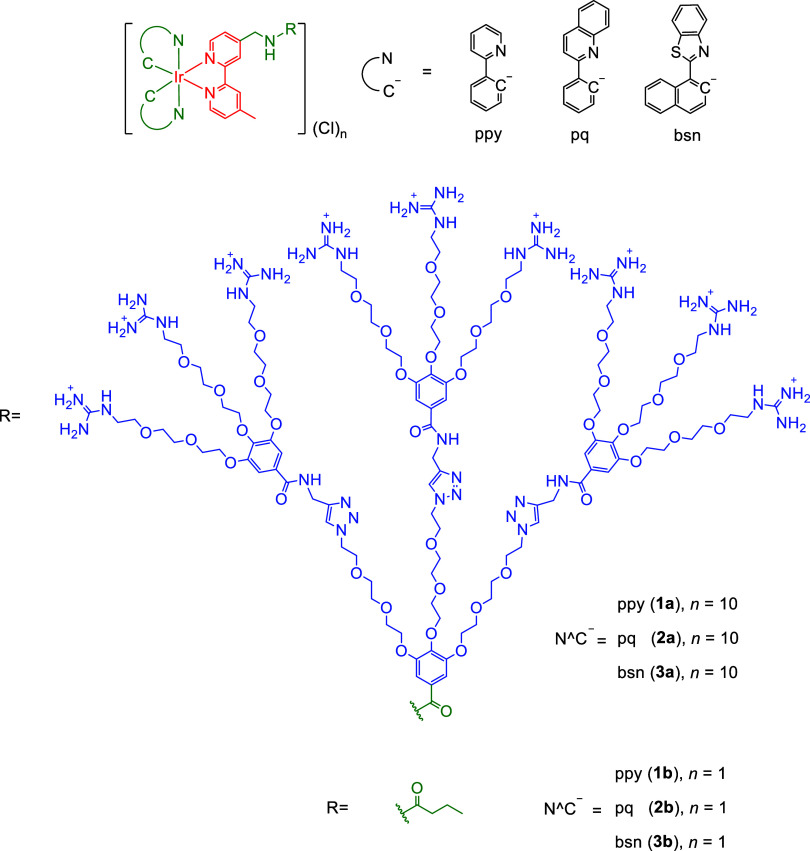
Structures of Complexes **1a**–**3a** and **1b**–**3b**

## Results and Discussion

### Synthesis

Cyclometalated iridium­(III) complexes were chosen for designing luminescent molecular glues because of their outstanding photophysical and photochemical characteristics, as well as their adaptable cellular behavior.^27–29^ We expect that the functionalization of iridium­(III) complexes with a dendritic guanidinium moiety can not only circumvent the problems of poor water solubility and high dark cytotoxicity, but also enhance their ability to form noncovalent interaction with biomolecules. Schemes 1 and 2 illustrate the synthetic procedure of the ligands and complexes, respectively. The ligand 4-(*N*-(3,4,5-tris­(2-(2-(2-azidoethoxy)­ethoxy)­ethoxy)­phenylcarbonyl)­aminomethyl)-4’-methyl-2,2’-bipyridine (bpy-Ph-(TEG-N_3_)_3_) was synthesized via the amide coupling reaction between 4-aminomethyl-4’-methyl-2,2’-bipyridine (bpy-CH_2_-NH_2_)^36^ and a dendron carrying a carboxylic acid group and three azide pendants (HOOC-Ph-(TEG-N_3_)_3_).^37^ The ligand bpy-Ph-(TEG-N_3_)_3_ was reacted with the precursor complexes [Ir_2_(N^C)_4_Cl_2_] (HN^C = Hppy, Hpq, and Hbsn) in CH_2_Cl_2_/MeOH to afford [Ir­(N^C)_2_(bpy-Ph-(TEG-N_3_)_3_)]­(Cl) (complexes **1**–**3**). Complexes **1a**–**3a** were synthesized through a copper-catalyzed “click” reaction of a dendron containing an alkyne moiety and three *tert*-butoxycarbonyl (Boc)-protected guanidinium pendants (alkyne-Ph-(TEG-GuBoc_2_)_3_)^20^ with complexes **1**–**3**, followed by the deprotection of the Boc groups using HCl. The ligand bpy-C4 was isolated from the reaction of bpy-CH_2_-NH_2_ with butyric acid. Subsequent reaction of the ligand with iridium­(III) dimers afforded complexes **1b**–**3b**. All the complexes were obtained as orange-yellow solids, and characterized by ESI-MS/MALDI-TOF-MS, ^1^H and ^13^C NMR, and IR spectroscopy. Complexes **1**–**3** and **1b**–**3b** displayed good solubility in polar organic solvents while showing poor water solubility. In contrast, the guanidinium complexes **1a**–**3a** were highly soluble in water and insoluble in solvents of lower polarity such as CH_2_Cl_2_.

**Scheme 1:**
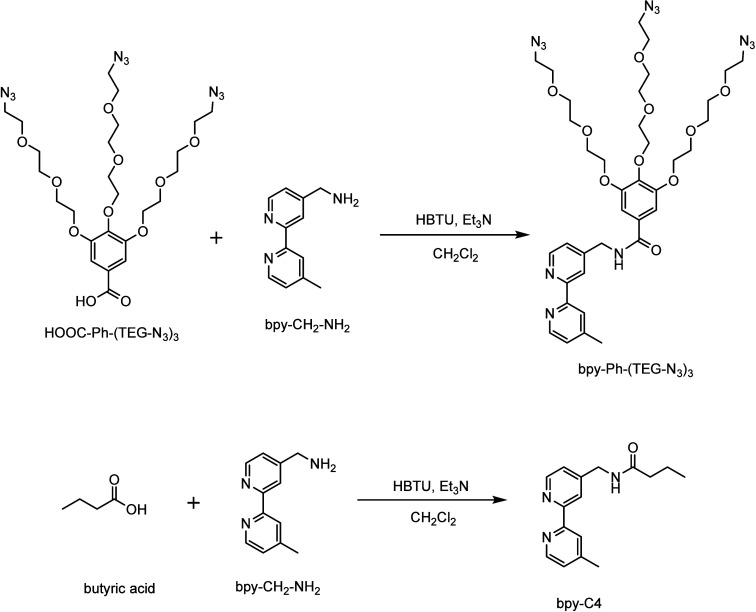
Synthetic Route of the Ligands bpy-Ph-(TEG-N_3_)_3_ and bpy-C4

**Scheme 2:**
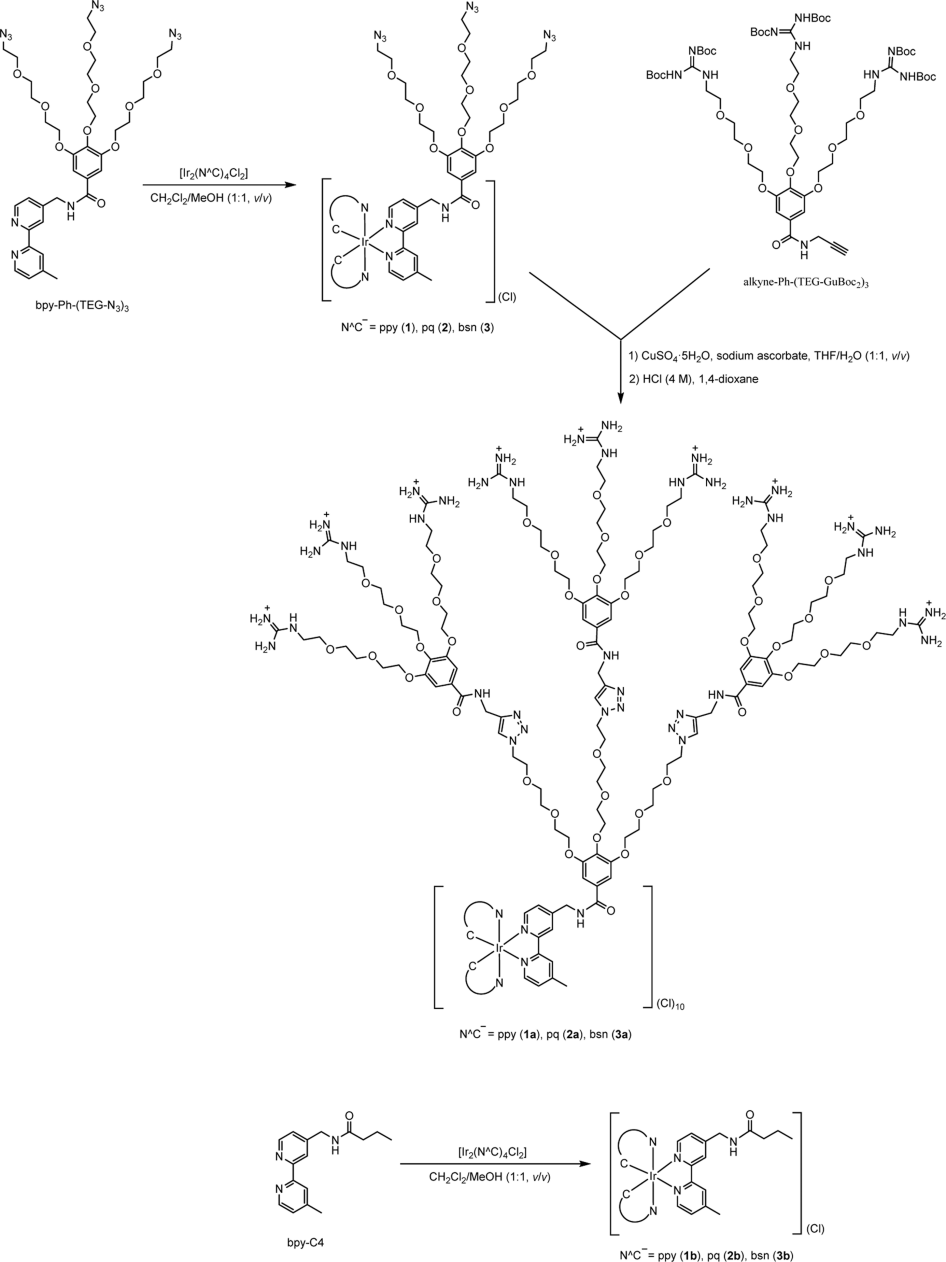
Synthetic Routes of Complexes **1**–**3**, **1a**–**3a**, and **1b**–**3b**

### Photophysical and Photosensitization Properties

The UV–vis absorption data of the complexes are provided in Table S1, while the spectra are depicted in Figures 1a, S1, and S2. Similar to other related mixed-ligand iridium­(III) diimine systems,^38–40^ complexes **1a**–**3a** and **1b**–**3b** exhibited strong intraligand (IL) (π → π*) (N^N and N^C) absorption characteristics at ca. 250–352 nm and less intense metal-to-ligand charge-transfer (MLCT) (dπ­(Ir) → π*­(N^N and N^C)) absorption at ca. 380–470 nm. Additionally, the much weaker tailing occurring at >480 nm was attributed to spin-forbidden MLCT (dπ­(Ir) → π*­(N^N and N^C)) transitions.

**Figure 1:**
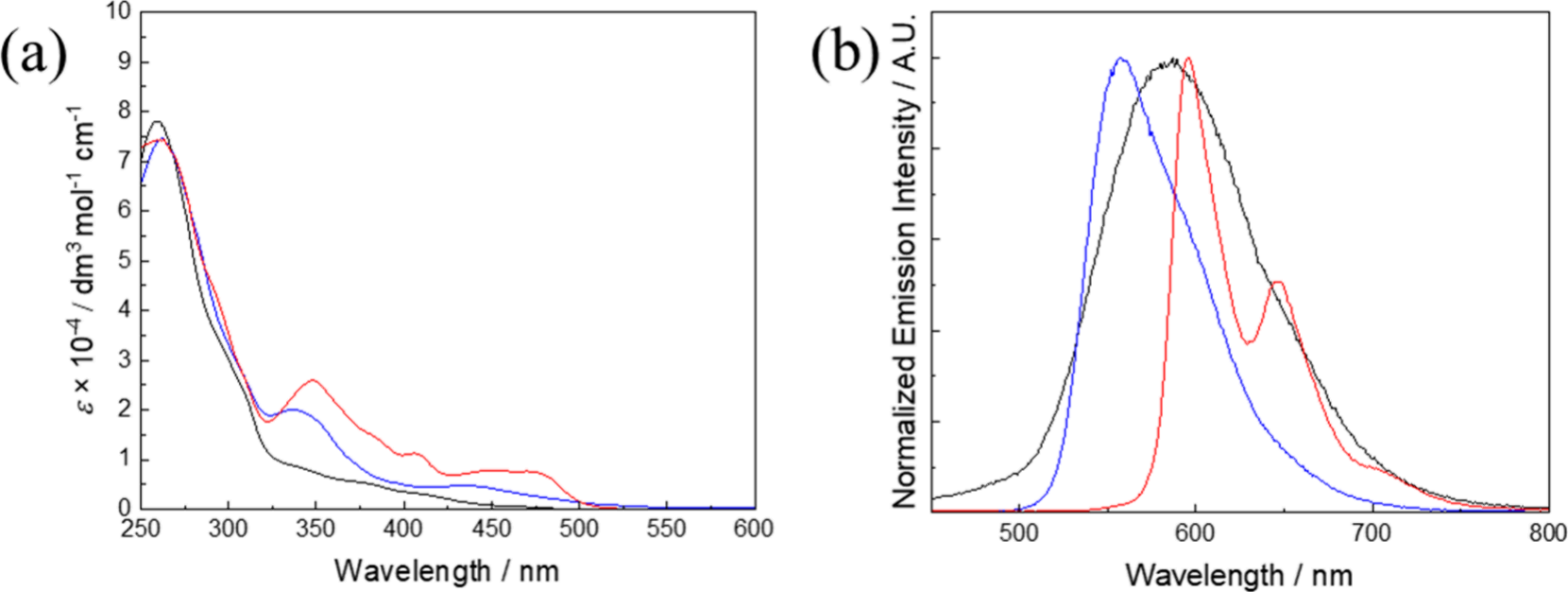
(a) UV–vis absorption spectra and (b) normalized emission spectra of complexes **1a** (black), **2a** (blue), and **3a** (red) in H_2_O at 298 K.

Excitation of all the complexes resulted in intense greenish-yellow to red emission (Table 1 and Figures 1b, S3, and S4). Among them, the ppy complexes (**1a** and **1b**) displayed a broad emission band with positive solvatochromic response (i.e., occurrence at lower energy in more polar H_2_O than in less polar MeOH) in solutions at 298 K. The emission maxima of the samples in alcohol glass (77 K) appeared at higher energy, indicative of an emissive state of charge-transfer character, possibily an admixture of ^3^MLCT (dπ­(Ir) → π*­(N^N and N^C))/triplet ligand-to-ligand charge-transfer (^3^LLCT) (π­(N^C) → π*­(N^N)).^38^ On the contrary, the pq (**2a** and **2b**) and bsn (**3a** and **3b**) complexes exhibited emission bands with vibronic structures and extended lifetimes in solutions at 298 K. Furthermore, the emission characteristics of these complexes were less affected by the polarity of the solvents, suggesting substantial ^3^IL (π → π*) (N^C) character in their excited states.^39,40^ Notably, the emission quantum yields of complexes **1a**–**3a** (Φ_em_ = 0.13–0.45 in MeOH, 0.02–0.37 in H_2_O; Table 1) were somewhat lower compared to those of the guanidinium-free complexes **1b**–**3b** (Φ_em_ = 0.14–0.58 in MeOH, 0.05–0.52 in H_2_O; Table 1). It is likely that the appended flexible guanidinium moieties enhanced the nonradiative deactivation efficiencies of complexes **1a**–**3a**.

**Table 1:** Photophysical Data of Complexes **1a**–**3a** and **1b**–**3b**

complex	medium (*T*/K)	λ_em_/nm^a^	τ_o_/μs^b^	Φ_em_ ^c^
**1a**	MeOH (298)	579	0.28	0.13
	H_2_O (298)	591	0.08	0.02
	glass^d^ (77)	512, 538 sh	4.07	
**2a**	MeOH (298)	561, 592 sh	2.62	0.45
	H_2_O (298)	567, 594 sh	2.00	0.37
	glass^d^ (77)	541, 586 sh	4.67	
**3a**	MeOH (298)	595 (max), 646, 708 sh	3.55	0.15
	H_2_O (298)	598 (max), 649, 708 sh	2.81	0.10
	glass^d^ (77)	590 (max), 610, 641, 663 sh, 703	5.11	
**1b**	MeOH (298)	588	0.32	0.14
	H_2_O^e^ (298)	597	0.15	0.05
	glass^d^ (77)	508, 536 sh	4.65	
**2b**	MeOH (298)	559, 589 sh	2.72	0.58
	H_2_O^e^ (298)	559, 589 sh	2.44	0.52
	glass^d^ (77)	543, 581 sh	4.73	
**3b**	MeOH (298)	594 (max), 645, 707 sh	3.21	0.18
	H_2_O^e^ (298)	596 (max), 647, 709 sh	2.46	0.12
	glass^d^ (77)	590 (max), 607, 641, 663 sh, 703	5.18	

a λ_ex_ = 350 nm.

b The emission lifetimes were recorded at the emission maxima (λ_ex_ = 355 nm).

c [Ru­(bpy)_3_]­Cl_2_ was used as a reference (Φ_em_ = 0.040 in air-saturated H_2_O, λ_ex_ = 455 nm).

d EtOH/MeOH (4:1, *v*/*v*).

e H_2_O/MeOH (4:1, *v*/*v*).

The ^1^O_2_ quantum yields (Φ_Δ_) of the complexes were measured in air-saturated MeOH. The ^1^O_2_ generation efficiencies of the guanidinium complexes **1a**–**3a** slightly decreased (Φ_Δ_ = 0.47–0.93; Table 2) compared to their guanidinium-free counterparts **1b**–**3b** (Φ_Δ_ = 0.64–0.97; Table 2), which could be due to the cage effect of the guanidinium moieties. The highest ^1^O_2_ quantum yields of the bsn complexes **3a** and **3b** are attributed to the longest emission lifetimes of these complexes (Table 1).

**Table 2:** ^1^O_2_ Quantum Yields of Complexes **1a**–**3a** and **1b**–**3b** in Air-Saturated MeOH at 298 K

complex	Φ_Δ_ ^a^
**1a**	0.47
**2a**	0.72
**3a**	0.93
**1b**	0.64
**2b**	0.82
**3b**	0.97

a [Ru­(bpy)_3_]­Cl_2_ was used as the standard (Φ_Δ_ = 0.73 in air-saturated MeOH).^41^

### Adhesion Properties of Complexes **1a**–**3a**

We examined the capability of the decacationic guanidinium complexes **1a**–**3a** as molecular glues. Initially, we investigated the adhesion strength between these complexes with proteins, using BSA, which has a negatively charged surface, as a model protein. The binding constants (*K*_b_) of complexes **1a**–**3a** with BSA were measured through emission titration experiments. The emission spectral traces and titration curves of complexes **1a**–**3a** against BSA are presented in Figures 2 and 3, respectively. Addition of BSA to complexes **1a**–**3a** (5 μM) in phosphate-buffered saline (PBS) increased the emission intensities of the solutions. This is ascribed to the enhanced rigidity and hydrophobic nature of the microenvironment of the complexes after binding to the protein molecule.^30–35^ Through fitting the fractions of bound complexes to the Hill equation, the *K*_b_ values for complexes **1a**–**3a** were determined to be 3.4, 5.5, and 7.1 × 10^5^ M^–1^, respectively. These values are comparable to previously reported guanidinium-based molecular glues (*K*_b_ = 6.3 × 10^5^ M^–1^),^15^ indicating that the adhesion properties of the guanidinium moieties did not change significantly after modification with the iridium­(III) complexes.

**Figure 2:**
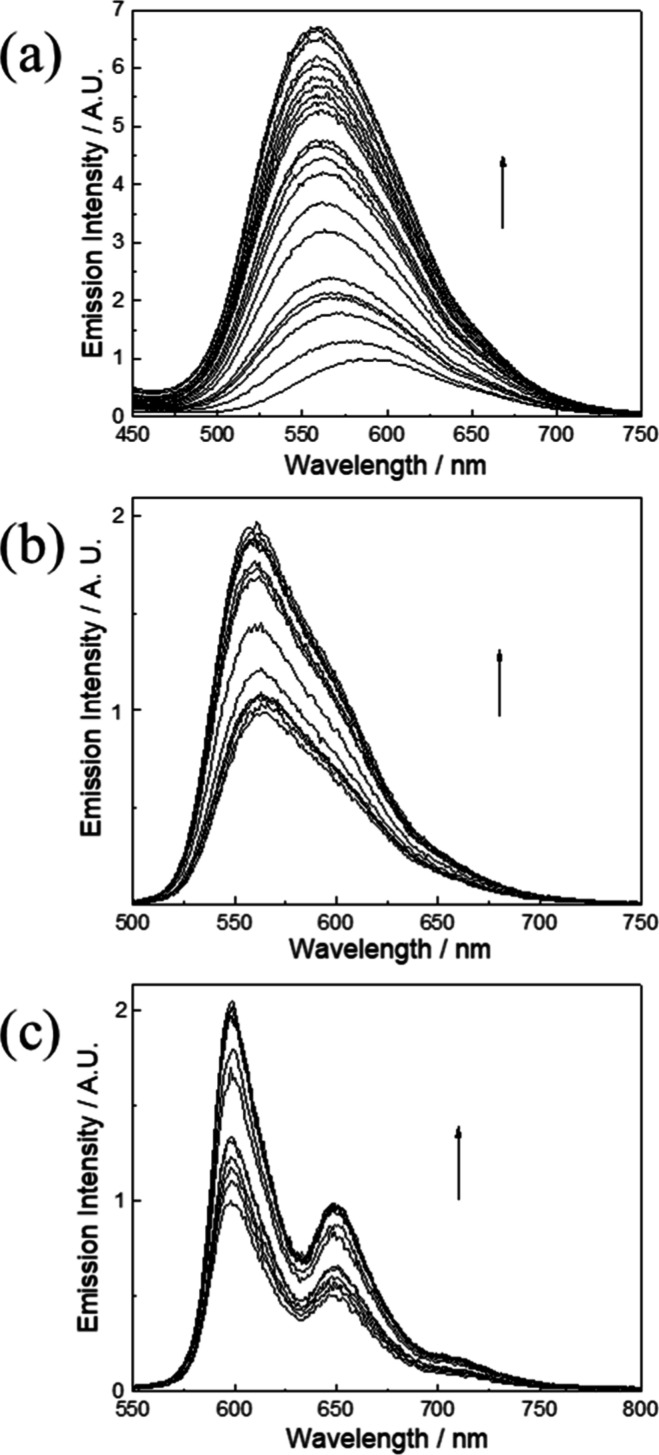
Emission spectral traces of complexes (a) **1a**, (b) **2a**, and (c) **3a** (5 μM) in PBS (pH 7.4) at 298 K upon the addition of BSA (0–20 μM for complex **1a**; 0–10 μM for complexes **2a** and **3a**).

**Figure 3:**
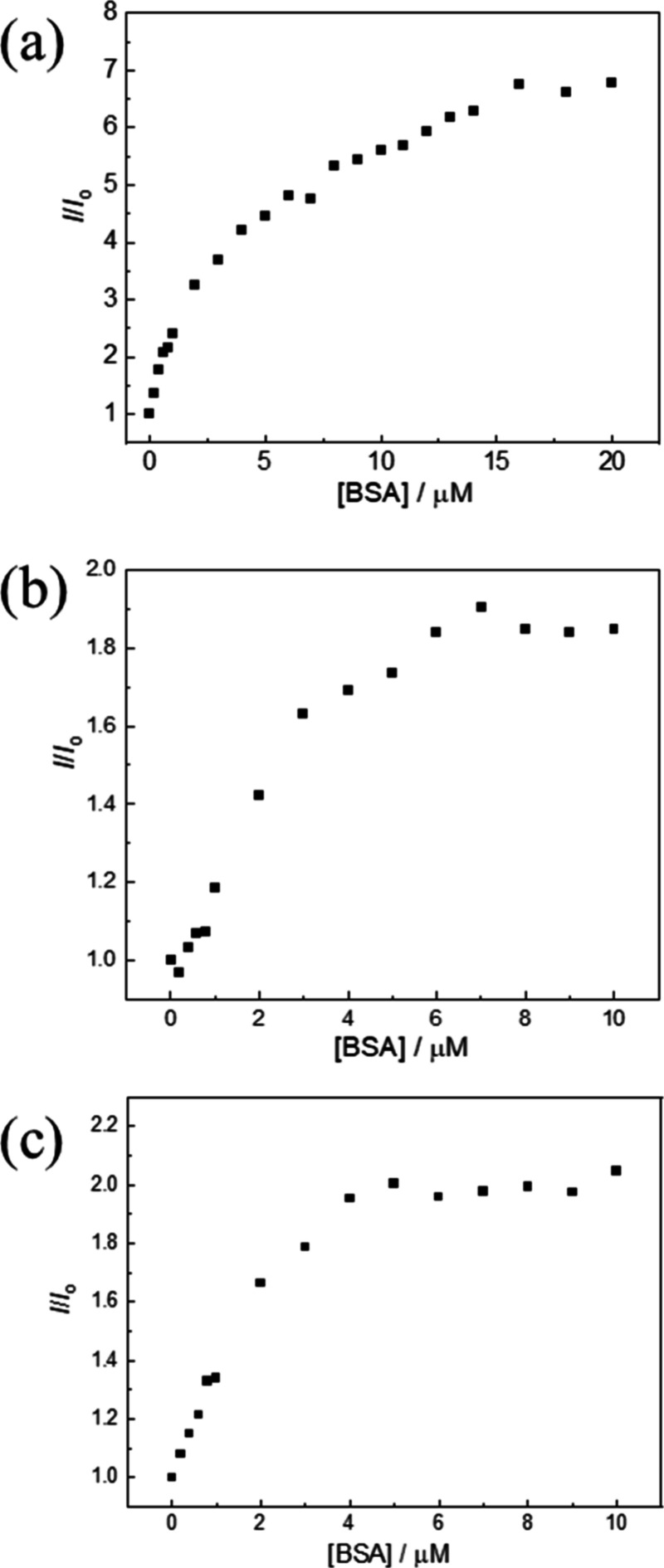
Emission titration curves of complexes (a) **1a**, (b) **2a**, and (c) **3a** (5 μM) with BSA (0–20 μM for complex **1a**; 0–10 μM for complexes **2a** and **3a**). *I*_o_ and *I* are the emission intensities of the complexes, measured at the maxima, in the absence and presence of BSA, respectively.

### Cellular Uptake and (Photo)­cytotoxicity Studies

We assessed the efficiencies of the uptake of complexes **1a**–**3a** and **1b**–**3b** by HeLa cells using inductively coupled plasma-mass spectrometry (ICP-MS). The complexes demonstrated effective cellular internalization after 2 h of incubation, with intracellular iridium levels ranked as follows: **3a** > **2a** > **1a** and **3b** > **2b** > **1b** (Table 3). This order corresponds to the lipophilicity of the ligands: bsn > pq > ppy. Notably, the cellular internalization of the guanidinium complexes **1a**–**3a** ([Ir] = 0.37–0.53 fmol) was lower than that of the guanidinium-free counterparts **1b**–**3b** ([Ir] = 0.47–0.94 fmol), likely due to decreased lipophilicity and increased molecular size resulting from the addition of a dendritic guanidinium group.

**Table 3:** Uptake of Complexes **1a**–**3a** and **1b**–**3b** by an Average HeLa Cell

complex	amount of complex/fmol
**1a**	0.37 ± 0.03
**2a**	0.42 ± 0.01
**3a**	0.53 ± 0.06
**1b**	0.47 ± 0.03
**2b**	0.64 ± 0.03
**3b**	0.94 ± 0.04

We then investigated the (photo)­cytotoxicity of the complexes toward HeLa cells by the 3-(4,5-dimethylthiazol-2-yl)-2,5-diphenyltetrazolium bromide (MTT) assay. Under dark conditions, the guanidinium complexes **1a**–**3a** displayed moderate cytotoxicity with IC_50_ values ranging from 11–12 μM (Table 4), which are comparable to those of the previously reported dendritic molecular glues (IC_50,dark_ = 11 μM).^19^ The similar dark cytotoxicity of complexes **1a**–**3a** suggests that the cytotoxic activity of these complexes mainly originates from the dendritic guanidinium moiety and is less dependent on the cyclometalating ligands. Upon irradiation, the cytotoxic effect of the complexes was remarkably enhanced, with IC_50_ values dropping sharply to as low as 0.27 μM (Table 4), most likely due to the effects of photoinduced formation of ^1^O_2_. The highest photocytotoxicity of complex **3a** should be associated with its highest cellular uptake (Table 3) and most efficient ^1^O_2_ generation (Φ_Δ_ = 0.93; Table 2). In contrast, the guanidinium-free complexes **1b**–**3b** generally exhibited higher cytotoxicity than the guanidinium complexes **1a**–**3a** in the dark and upon irradiation (Table 4) as a result of the relatively higher cellular uptake (Table 3) and ^1^O_2_ generation efficiencies (Table 2) of the guanidinium-free complexes.

**Table 4:** (Photo)­cytotoxic Effects of Complexes **1a**–**3a** and **1b**–**3b** toward HeLa Cells under Dark or Light Conditions (λ_ex_ = 450 nm, 15.5 mW cm^–2^, 10 min)

complex	IC_50,dark_/μM	IC_50,light_/μM	photocytotoxicity index^a^
**1a**	11 ± 2	7.1 ± 0.2	1.5
**2a**	11 ± 1	1.8 ± 0.1	6.1
**3a**	12 ± 2	0.27 ± 0.05	44.4
**1b**	25 ± 3	0.73 ± 0.01	34.2
**2b**	1.6 ± 0.1	0.075 ± 0.021	21.3
**3b**	0.81 ± 0.01	0.052 ± 0.014	15.6

a Photocytotoxicity index is defined as IC_50,dark_/IC_50,light_.

### Live-Cell Confocal Imaging

We examined the localization of the complexes in HeLa cells using laser-scanning confocal microscopy (LSCM). Upon incubation with complexes **1a**–**3a** (5 μM, 2 h), intense emission was observed in the cell interior (Figure 4). Costaining experiments with commercially available organelle stains such as MitoTracker Deep Red, ER-Tracker Green, and LysoTracker Deep Red revealed the specific localization of complexes **1a** and **3a** in the mitochondria and ER, respectively (Pearson’s correlation coefficient (PCC) = 0.81 and 0.78, respectively) (Figure 4a,c), but not the other organelles (Figures S5–S7). Interestingly, cells treated with complex **2a** displayed punctate staining in the nuclear region, likely originating from the nucleoli (Figure 4b). To verify this, we costained HeLa cells treated with complex **2a** with a fibrillarin antibody (2 μL mL^–1^, 18 h) and employed Alexa Fluor 647-conjugated antirabbit IgG (2 μL mL^–1^, 1 h) as the secondary antibody. Despite the small PCC value (0.36) due to the differential staining of the cytoplasm and nucleoplasm by complex **2a** and fibrillarin, respectively (Figure 4b), the strong overlap of the granular staining of complex **2a** with fibrillarin inside the nucleus indicates the interesting nucleoli-targeting capability of complex **2a**. It is noteworthy that the intracellular localization of the three guanidinium complexes depends heavily on the nature of the cyclometalating ligands. Conversely, HeLa cells exposed to the guanidium-free complexes **1b**–**3b** (5 μM, 2 h) exhibited intense perinuclear emission (Figure 5). Costaining studies using MitoTracker Deep Red (100 nM, 20 min) confirmed the enrichment of the complexes in the mitochondria (PCC values ranging from 0.89 to 0.95). The high specificity of complexes **1b**–**3b** for mitochondria is attributed to their significant lipophilicity and monocationic charge.^42–46^

**Figure 4:**
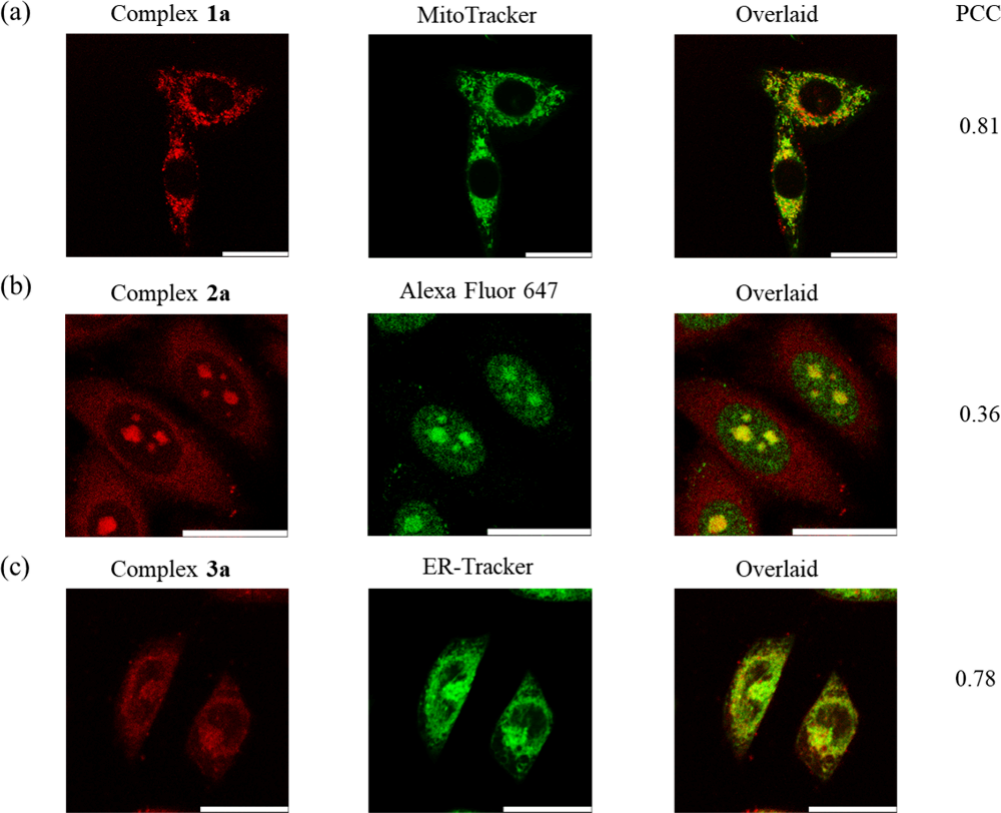
LSCM images of (a) live HeLa cells incubated with complex **1a** and MitoTracker Deep Red; (b) fixed HeLa cells incubated with complex **2a**, fibrillarin antibody, and Alexa Fluor 647-conjugated antirabbit IgG antibody; and (c) live HeLa cells incubated with complex **3a** and ER-Tracker Green. Scale bar = 25 μm.

**Figure 5:**
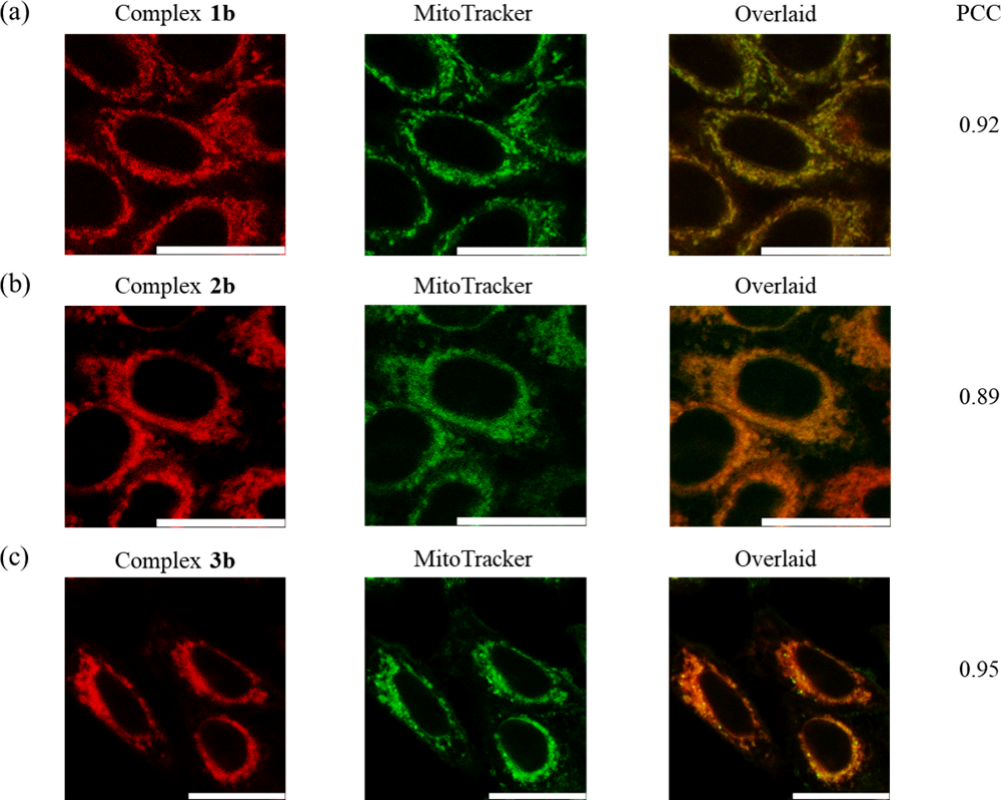
LSCM images of live HeLa cells incubated with complexes (a) **1b**, (b) **2b**, or (c) **3b** and MitoTracker Deep Red. Scale bar = 25 μm.

### Preparation and Characterization of GSH-Responsive BSA Nanoparticles

Nanoparticles made from proteins can be easily produced using BSA and have been widely employed as a drug delivery system for transporting drugs such as DOX into cells.^47–49^ We constructed DOX-loaded GSH-responsive BSA nanoparticles (DOX/^SS^BNPs) based on a reported desolvation method with minor modifications.^50,51^ A solution of BSA and DOX·HCl in H_2_O was adjusted to pH 9 with NaOH. Addition of EtOH induced the formation of nanoparticles. Subsequent cross-linking of the nanoparticles with 3,3′-dithiobispropanoic acid bis­(*N*-hydroxysuccinimide ester) (DSP) led to the formation of DOX/^SS^BNPs (Scheme 3). The nanoparticles were purified by centrifugation and resuspended in H_2_O. The drug loading (DL) and encapsulation efficiencies (EE) were found to be 2.7 and 27.2%, respectively. The concentration of DOX loaded in the nanoparticles was estimated to be 0.5 μg mL^–1^ at [DOX/^SS^BNPs] = 0.2 mg mL^–1^. Additionally, DOX-free BSA nanoparticles (^SS^BNPs) were prepared, using a similar method without the addition of DOX, for comparison studies. As revealed by transmission electron microscopy (TEM), both DOX/^SS^BNPs and ^SS^BNPs exhibited a monodispersed and spherical morphology with nanoscale dimensions (Figure 6a). Dynamic light scattering (DLS) and zeta potential analyses were employed to measure the size and surface charge of DOX/^SS^BNPs and ^SS^BNPs. The average hydrodynamic diameters were found to be 179.7 nm for DOX/^SS^BNPs and 143.1 nm for ^SS^BNPs, both exhibiting a narrow distribution with a polydispersity index (PDI) of less than 0.2 (Table S2 and Figure 6b). Additionally, both DOX/^SS^BNPs and ^SS^BNPs had a negatively charged surface (−20.2 and −28.6 mV, respectively; Table S2). Upon photoexcitation at 480 nm, DOX/^SS^BNPs displayed a vibronically structured emission band (Figure 6c, red), characteristic of DOX fluorescence (Figure S8), while no emission was detected for ^SS^BNPs (Figure 6c, black). These findings confirm that DOX was successfully loaded into the BSA nanoparticles without significantly affecting the size, morphology, and surface charge of the nanoparticles.

**Scheme 3:**
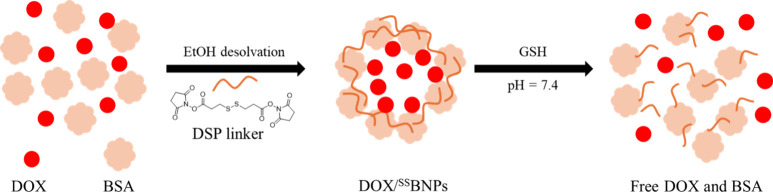
Schematic Illustration of the Formation of DOX/^SS^BNPs and Their Disassembly Triggered by GSH

**Figure 6:**
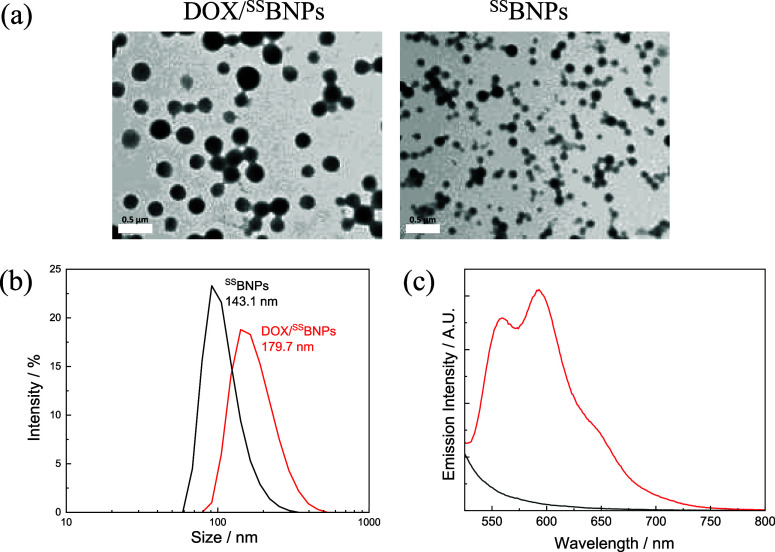
(a) TEM images of DOX/^SS^BNPs (left) and ^SS^BNPs (right). Scale bar = 500 nm. (b) DLS analysis of DOX/^SS^BNPs (0.2 mg mL^–1^) (red) and ^SS^BNPs (0.2 mg mL^–1^) (black) in H_2_O. (c) Emission spectra of DOX/^SS^BNPs (red) and ^SS^BNPs (black) in H_2_O at 298 K upon irradiation at 480 nm.

### Preparation of Iridium­(III) Complex-Modified DOX/^SS^BNPs and Their GSH-Triggered Disassembly

We first examined the interactions between the guanidinium complexes and DOX/^SS^BNPs using complex **1a** as a model compound. As revealed by the zeta potential measurements, the surface charge of DOX/^SS^BNPs (0.2 mg mL^–1^) (zeta potential = −20.2 mV) substantially increased upon the addition of complex **1a**, reaching a zeta potential of +19 mV at [complex **1a**] = 10 μM (Figure 7a). These indicate that functionalization of DOX/^SS^BNPs with the decacationic guanidinium complexes effectively reversed the surface charge from negative to positive, a crucial factor in facilitating their cellular uptake across the plasma membrane. Thus, the concentration of the guanidinium complexes used to modify DOX/^SS^BNPs and ^SS^BNPs (0.2 mg mL^–1^) was fixed at 10 μM to afford the corresponding nanoparticles Ir-DOX/^SS^BNPs and Ir-^SS^BNPs for the subsequent experiments.

**Figure 7:**
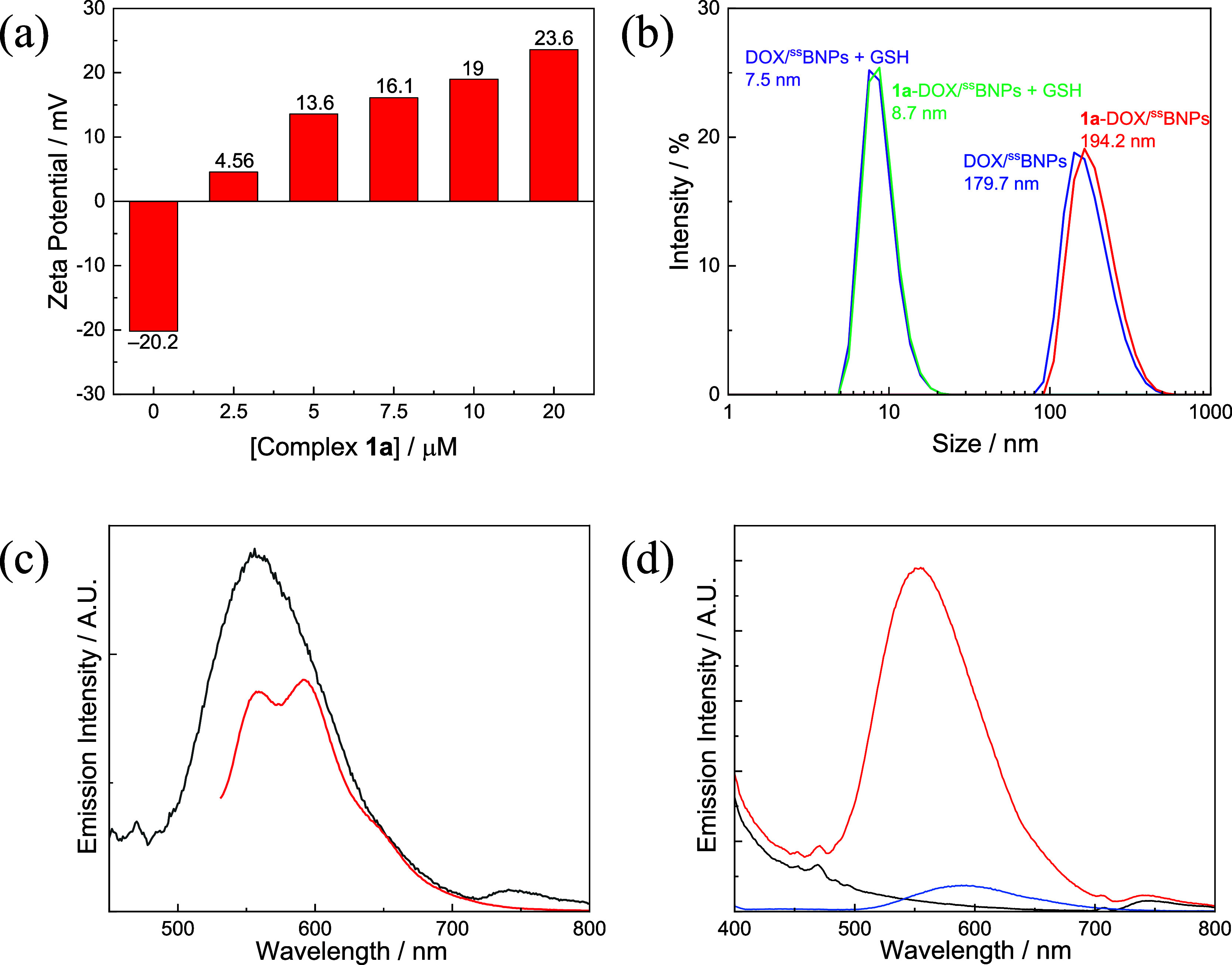
(a) Zeta potential histogram of DOX/^SS^BNPs (0.2 mg mL^–1^) treated with complex **1a** at different concentrations (0–10 μM) in H_2_O at 298 K. (b) DLS analysis of **1a**-DOX/^SS^BNPs ([Ir] = 10 μM, [DOX/^SS^BNPs] = 0.2 mg mL^–1^) and DOX/^SS^BNPs (0.2 mg mL^–1^) in H_2_O in the absence and presence of GSH (10 mM) upon treatment at 298 K for 1 h. Red: **1a**-DOX/^SS^BNPs; Blue: DOX/^SS^BNPs; Green: **1a**-DOX/^SS^BNPs + GSH; Purple: DOX/^SS^BNPs + GSH. (c) Emission spectra of **1a**-DOX/^SS^BNPs ([Ir] = 10 μM, [DOX/^SS^BNPs] = 0.2 mg mL^–1^) in PBS (pH 7.4) at 298 K upon irradiation at 350 (black) and 480 nm (red). (d) Emission spectra of **1a**-^SS^BNPs ([Ir] = 10 μM, [^SS^BNPs] = 0.2 mg mL^–1^) (red), ^SS^BNPs (0.2 mg mL^–1^) (black), and complex **1a** (10 μM) (blue) in PBS (pH 7.4) at 298 K upon irradiation at 350 nm.

After modification with complex **1a**, the size of DOX/^SS^BNPs increased slightly from 179.7 (Figure 7b, blue) to 194.2 nm (Figure 7b, red). Upon photoexcitation at 350 nm, **1a**-DOX/^SS^BNPs showed a strong and broad emission band at 555 nm (Figure 7c, black) with a lifetime of 386 ns, attributable to the iridium­(III)-based phosphorescence. However, upon irradiation at 480 nm, a lower-energy emission band with two maxima at 561 and 591 nm was observed (Figure 7c, red), which should be the fluorescence of DOX in the nanoparticles. We also studied the emission properties of the DOX-free **1a**-^SS^BNPs and compared them to those of the unmodified ^SS^BNPs and complex **1a**. Similarly, **1a**-^SS^BNPs displayed the characteristic emission of complex **1a** upon irradiation at 350 nm (Figure 7d, red). Importantly, the emission maximum was hypsochromically shifted from 593 to 555 nm compared to complex **1a** alone (Figure 7d, blue), accompanied by substantial emission enhancement (*I*/*I*_o_ = 13.1) and lifetime elongation (from 75 to 503 ns). These photophysical changes indicate the relatively hydrophobic local environment of complex **1a** provided by ^SS^BNPs. In contrast, ^SS^BNPs exhibited very weak emission (Figure 7d, black), which is attributed to fluorescence of the tryptophan and tyrosine residues in BSA.

Importantly, in the presence of GSH (10 mM), the hydrodynamic diameters of **1a**-DOX/^SS^BNPs and DOX/^SS^BNPs decreased significantly from 194.2 and 179.7 nm (Figure 7b, red and blue) to 8.7 and 7.5 nm (Figure 7b, green and purple), respectively. TEM analysis also revealed efficient disassembly of **1a**-DOX/^SS^BNPs in the presence of GSH (Figure 8). The decrease in size can be ascribed to the efficient DOX release upon the reductive cleavage of the disulfide bonds in the BSA nanoparticles.

**Figure 8:**
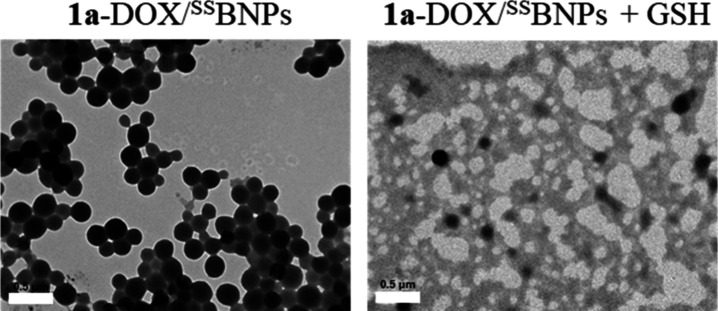
TEM images of **1a**-DOX/^SS^BNPs ([Ir] = 10 μM, [DOX/^SS^BNPs] = 0.2 mg mL^–1^) in H_2_O in the absence (left) and presence (right) of GSH (10 mM) upon treatment at 298 K for 1 h. Scale bar = 500 nm.

### Intracellular Delivery of DOX by Ir-DOX/^SS^BNPs and DOX/^SS^BNPs

The intracellular delivery of DOX by Ir-DOX/^SS^BNPs and DOX/^SS^BNPs was studied by LSCM. HeLa cells were incubated with **1a**-DOX/^SS^BNPs ([Ir] = 10 μM, [DOX/^SS^BNPs] = 0.2 mg mL^–1^) for different periods. After incubation for 1 h, the emission of complex **1a** and fluorescence of DOX were both detected in the cytoplasm of HeLa cells, while almost no luminescence was observed from the nucleus (Figure 9a–c). When the incubation time was increased to 2 and 4 h, stronger emission was observed in the cell interior (Figure 9d–i) due to increased cellular uptake of **1a**-DOX/^SS^BNPs. Notably, the fluorescence intensity of DOX in the nucleus was enhanced (Figure 9b, e, and h), indicating that DOX was efficiently released in the cytoplasm and subsequently transported to the nucleus. The negligible overlap of the emission of complex **1a** and DOX (Figure 9c, f, and i) also supports the release of DOX from the nanoparticles. In contrast, HeLa cells treated with DOX/^SS^BNPs for 4 h showed extremely weak emission in the nucleus (Figure S9a–c), indicating that only a small amount of DOX/^SS^BNPs was internalized into the cells. Furthermore, cells treated with complex **1a** only displayed strong emission in the cytoplasm upon photoexcitation at 405 nm, and no emission was detected upon 488 nm excitation (Figure S9d–f), confirming that there was no spectral interference between DOX and complex **1a**. Collectively, these results indicate that the presence of guanidinium complexes substantially facilitated the cellular uptake of Ir-DOX/^SS^BNPs, as the decacationic complexes can reverse the surface charge of BSA nanoparticles from negative to positive (Figure 7a), allowing their interaction with the negatively charged plasma membrane. After cellular uptake, the nanoparticles were disassembled due to the sensitivity of the disulfide bonds to intracellular GSH, releasing the iridium­(III) complexes and DOX from the nanoparticles. The released DOX gradually translocated from the cell cytoplasm to the nucleus where it exerted its anticancer effect, while the iridium­(III) complexes were retained in the cytoplasmic region.

**Figure 9:**
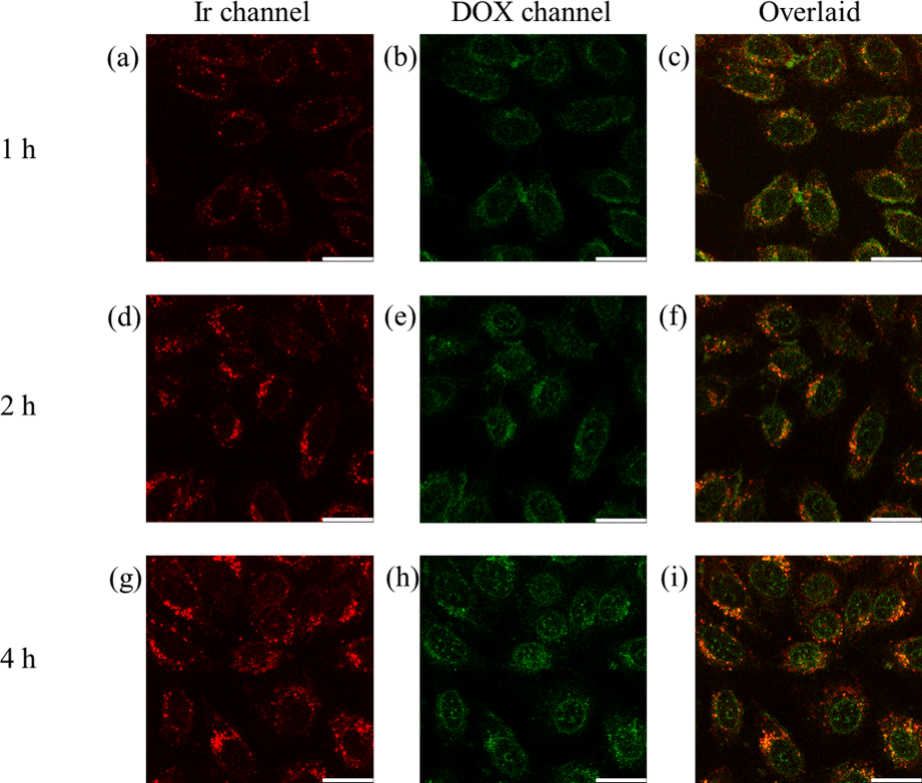
LSCM images of live HeLa cells incubated with **1a**-DOX/^SS^BNPs at 37 °C for (a–c) 1, (d–f) 2, and (g–i) 4 h. Scale bar = 25 μm.

### Combination Therapy

To evaluate the application of Ir-DOX/^SS^BNPs in chemo-photodynamic therapy, the (photo)­cytotoxic effects of Ir-DOX/^SS^BNPs were evaluated by the MTT assay. We selected **3a**-DOX/^SS^BNPs as a model in this study due to the high ^1^O_2_ generation quantum yield of complex **3a** (Φ_Δ_ = 0.93; Table 2). HeLa cells were treated with **3a**-DOX/^SS^BNPs at various concentrations (where a fixed [Ir]/[DOX] ratio of 10.9 was maintained) at 37 °C for 4 h, incubated in the dark or irradiation at 450 nm for 10 min (light dose = 15.5 mW cm^–2^), and subsequently maintained in the dark for another 20 h. While free DOX showed high dark cytotoxicity toward HeLa cells (IC_50,dark_ = 3.7 μg mL^–1^; Figure S10, red), both **3a**-DOX/^SS^BNPs (Figure 10, blue) and DOX/^SS^BNPs (Figure S10, black) displayed remarkably lower cytotoxic activity under the same conditions. The viability of HeLa cells treated with **3a**-DOX/^SS^BNPs (Figure 10, blue) was also generally higher than that with complex **3a** (Figure S11) at the same [Ir], indicating the higher biocompatibility of **3a**-DOX/^SS^BNPs. Importantly, when the **3a**-DOX/^SS^BNPs-treated cells were exposed to irradiation, the cell viability dropped significantly, for example, from >95 to <5% at [Ir] = 0.625 μM (Figure 10).

**Figure 10:**
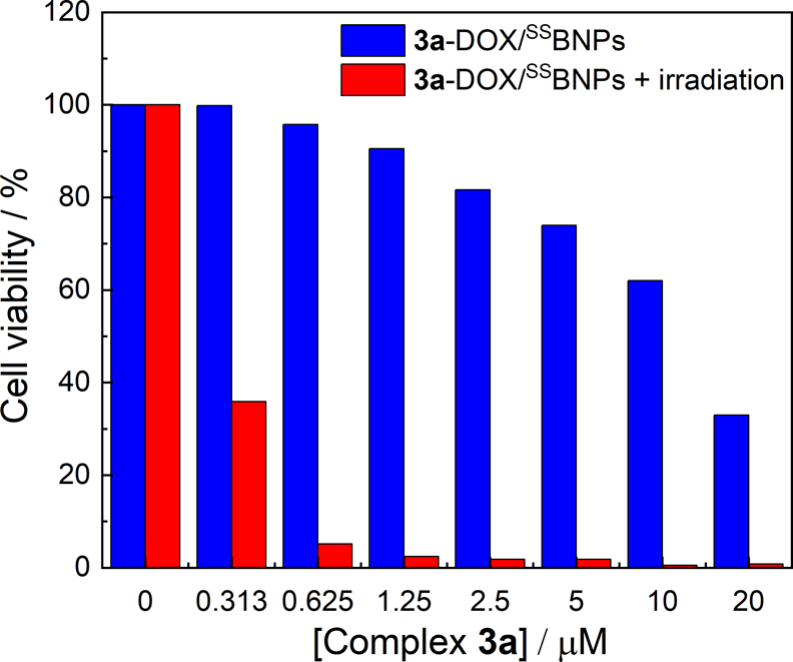
Viability of HeLa cells exposed to varying concentrations of **3a**-DOX/^SS^BNPs at 37 °C for 4 h, incubated in the dark (blue) or exposed to 450 nm light (red) for 10 min.

We investigated the mode of cell death induced by **1a**-DOX/^SS^BNPs with an Annexin V/propidium iodide **(**PI) assay. As illustrated in Figure 11, cells treated with **1a**-DOX/^SS^BNPs ([Ir] = 10 μM, [DOX/^SS^BNPs] = 0.2 mg mL^–1^) in the dark showed a high percentage of necrotic cells (Q1, 15.4%), which is much greater than that observed in DOX/^SS^BNPs (0.2 mg mL^–1^)-treated cells (7.79%). This increase is attributed to the more effective cellular uptake of **1a**-DOX/^SS^BNPs, enhancing the intracellular delivery of DOX. Importantly, when **1a**-DOX/^SS^BNPs-treated cells were exposed to irradiation, both apoptotic and necrotic cells were detected, with apoptosis (Q3, 25.0%) being the predominant cell death pathway due to the effective ^1^O_2_ generation by complex **1a** (Table 2), and the percentage of necrotic cells (Q1) changed from 15.4 to 5.30% upon irradiation. While complex **1a** (10 μM)-treated cells also showed a significant increase in apoptotic cells (Q3, from 0.66 to 21.3%) upon irradiation, the necrotic cell populations remained very low (Q1, 2.60 and 1.84% without and with irradiation, respectively). Thus, the efficient necrotic and apoptotic cell death caused by DOX and ^1^O_2_ generated by the excited state of complex **1a**, respectively, can highlight the potential of Ir-DOX/^SS^BNPs in chemo-photodynamic therapy.

**Figure 11:**
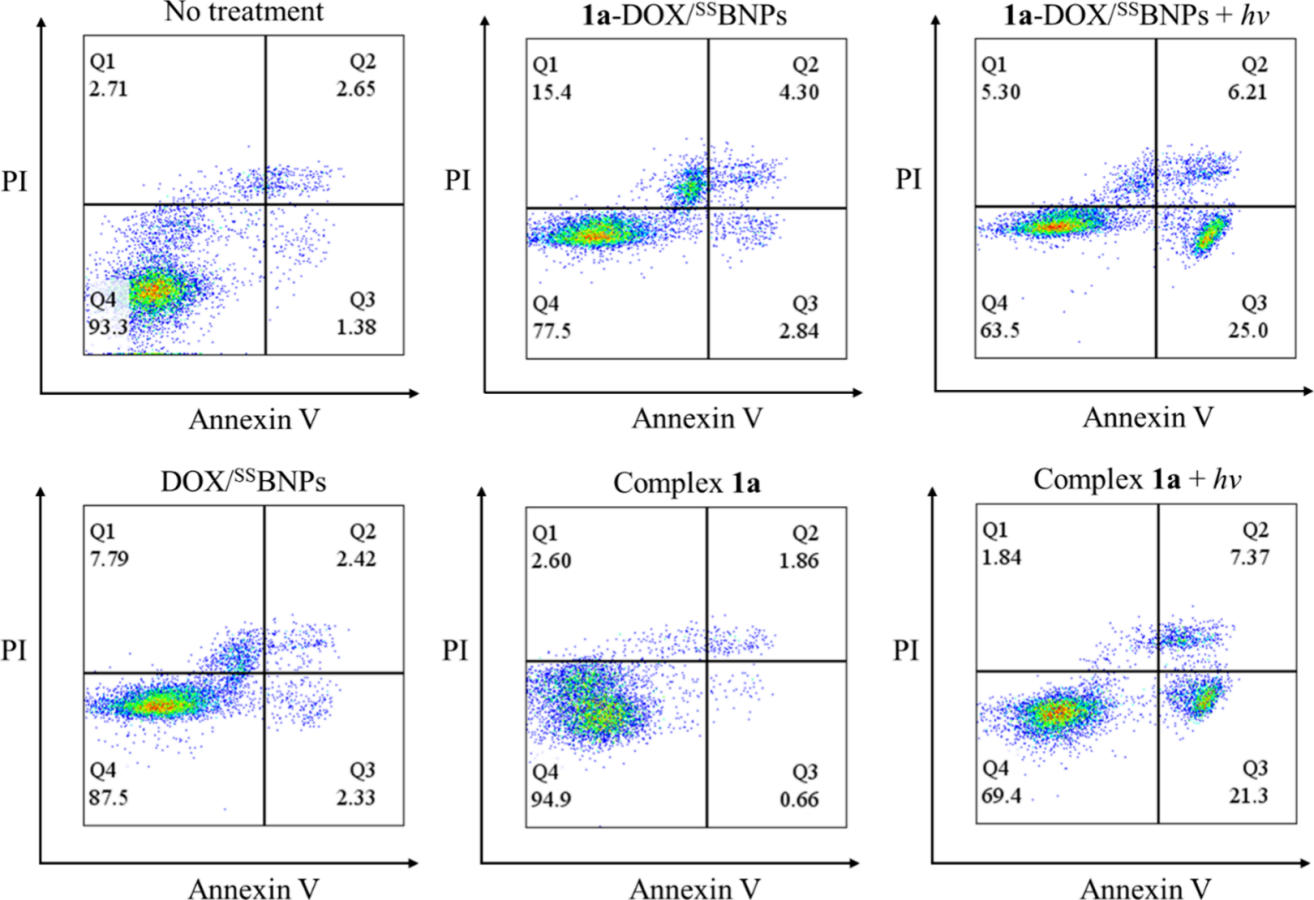
Flow cytometric analysis of HeLa cells treated with **1a**-DOX/^SS^BNPs, DOX/^SS^BNPs, or complex **1a** and incubated in the dark or irradiated at 450 nm.

## Conclusions

In this work, three luminescent molecular glues derived from iridium­(III) complexes appended with a dendritic guanidinium unit were developed. Their photophysics, photochemistry, and cellular uptake characteristics were investigated. These complexes displayed intense greenish-yellow to red emission and high ^1^O_2_ generation efficiencies. Upon cellular uptake, the complexes specifically localized in various cellular components including the mitochondria, nucleoli, and ER, controlled by the cyclometalating ligands. Subsequent exposure of the stained cells to continuous photoirradiation led to significant cell death with IC_50_ down to 0.27 μM. Notably, the introduction of a dendritic guanidinium moiety not only increased the water solubility of the complexes but also endowed them with strong capability to form multiple salt bridges with anionic molecules. Thus, the guanidinium complexes were further utilized as molecular glues for the development of iridium­(III) complex-modified, DOX-loaded, GSH-responsive BSA nanoparticles. Remarkably, functionalization of DOX/^SS^BNPs with the guanidinium complexes changed the negative surface charge of the nanoparticles to positive without interfering with the release of DOX when GSH was present. These modifications enhanced the cellular internalization of the nanoparticles, facilitating the delivery of DOX to the nucleus to exert its anticancer effect. Additionally, **3a**-DOX/^SS^BNPs were utilized for combined chemo-photodynamic therapy due to the high ^1^O_2_ generation efficiency of complex **3a**. When the cells were treated with **3a**-DOX/^SS^BNPs ([Ir] = 0.625 μM, [DOX/^SS^BNPs] = 0.0125 mg mL^–1^) and light irradiation, their viability dropped significantly from >95 to <5%. These results highlight that the iridium­(III) polyguanidinium complexes can not only serve as bioimaging regents and photocytotoxic agents, but also molecular glues for various biological and biomedical applications.

## Experimental Section

### Materials

All solvents were of analytical reagent grade and purified according to standard procedures.^52^ *N*,*N*-Diisopropylethylamine, 2-(1*H*-benzotriazole-1-yl)-1,1,3,3-tetramethyluronium hexafluorophosphate (HBTU), anhydrous MgSO_4_, butyric acid, CuSO_4_·5H_2_O, sodium ascorbate, 1,4-dioxane solution of HCl (4 M), paraformaldehyde, and methylene blue were purchased from Acros. 4,4’-Dimethyl-2,2’-bipyridine, SeO_2_, Na_2_S_2_O_5_, IrCl_3_·3H_2_O, Hppy, Hpq, BSA, doxorubicin hydrochloride (DOX·HCl), GSH, and MTT were purchased from Sigma-Aldrich. The DSP linker was purchased from Energy. All these chemicals were used without further purification. The ligands 4-carboxaldehyde-4’-methyl-2,2’-bipyridine (bpy-CHO),^39^ bpy-CH_2_-NH_2_,^36^ HOOC-Ph-(TEG-N_3_)_3_,^37^ alkyne-Ph-(TEG-GuBoc_2_)_3_,^20^ Hbsn,^40^ and iridium­(III) dimers [Ir_2_(N^C)_4_Cl_2_] (HN^C = Hppy, Hpq, and Hbsn)^53^ were prepared according to literature procedures. All buffer components were of biological grade and used as received. Autoclaved Milli-Q water was used for the preparation of the aqueous solutions. Human cervix carcinoma HeLa cells were obtained from American Type Culture Collection. Dulbecco’s modified Eagle’s medium (DMEM), fetal bovine serum (FBS), PBS, trypsin-EDTA, penicillin/streptomycin, MitoTracker Deep Red, ER-Tracker Green, LysoTracker Deep Red, triton X-100, fibrillarin antibody, Alexa Fluor 647-conjugated antirabbit IgG antibody, Alexa Fluor 647–Annexin V conjugate, Annexin V binding buffer, and PI were purchased from Invitrogen.

### Physical Measurements and Instrumentation

^1^H and ^13^C NMR spectra were recorded on a Bruker AVANCE III 300, 400, or 600 MHz NMR spectrometer at 298 K using deuterated solvents. Chemical shifts (δ, ppm) were reported relative to tetramethylsilane (TMS). Positive-ion ESI mass spectra were recorded on a PerkinElmer Sciex API 3200MD mass spectrometer at 298 K. MALDI-TOF mass spectra of the samples were recorded on an Applied Biosystems 4800 Plus MALDI TOF/TOF Analyzer. IR spectra of the samples in KBr pellets were recorded in the range of 4000–400 cm^–1^ using a PerkinElmer FTIR–1600 spectrometer. Electronic absorption spectra were recorded on an Agilent 8453 diode array spectrophotometer. Steady-state emission spectra were obtained on a HORIBA FluoroMax-4 spectrofluorometer. Unless specified otherwise, all solutions for photophysical studies were degassed with at least four successive freeze–pump–thaw cycles and stored in a 10 cm^3^ round bottomed flask equipped with a sidearm 1 cm fluorescence cuvette and sealed from the atmosphere by a Rotaflo HP6/6 quick-release Teflon stopper. Emission quantum yields were measured by the optically dilute method^54^ using an air-saturated aqueous solution of [Ru­(bpy)_3_]­Cl_2_ (Φ_em_ = 0.040, λ_ex_ = 455 nm)^55^ as the standard solution. The concentrations of the standard and sample solutions were adjusted until the absorbance at the excitation wavelength (455 nm) was 0.1. Emission lifetimes were measured on an Edinburgh Instruments LP920 laser flash photolysis spectrometer using the third harmonic output (355 nm; 6–8 ns fwhm pulse width) of a Spectra-Physics Quanta-Ray Q-switched LAB-150 pulsed Nd:YAG laser (10 Hz) as the excitation source. DLS and zeta potential measurements were performed at 298 K using a Malvern model Zetasizer Nano ZS particle size analyzer equipped with a 532 nm frequency doubled diode-pumped solid-state laser light source. The results were presented as number-based distributions.

### Determination of ^1^O_2_ Generation Quantum Yields (Φ_Δ_)

The Φ_Δ_ values of the complexes were determined by directly measuring the ^1^O_2_ fluorescence at 1,270 nm using an FLS-980 spectrofluorometer.^56^ All the complexes were dissolved in air-saturated MeOH with absorbance at 450 nm adjusted to 0.1. A 1,000 nm long-pass filter was placed between the sample solution and the NIR PMT detector to avoid high-order diffraction from the visible light. The Φ_Δ_ values were determined by comparing the integrated fluorescence intensity of ^1^O_2_ generated by excitation of [Ru­(bpy)_3_]­Cl_2_ at 450 nm (Φ_Δ_ = 0.73 in air-saturated MeOH).^41^

### Emission Titrations

The *K*_b_ values were determined using the Hill equation by plotting the binding fractions (θ) of complexes **1a**–**3a** as a function of BSA concentration. These binding fractions were calculated using the expression (*I* – *I*_o_)/(*I*_sat_ – *I*_o_), where *I*_o_ and *I* are the emission intensities of the complexes in the absence and presence of BSA, respectively, and *I*_sat_ is the emission intensity at saturation. The emission intensities of complexes **1a**–**3a** were monitored at the emission maxima. The resulting data were fitted to the Hill equation:

$$\theta =\frac{{\left[\mathrm{BSA}\right]}^{n}}{{\left[\mathrm{BSA}\right]}^{n}+{K_{d}}^{n}}$$

where [BSA] is the BSA concentration, *K*_d_ (= 1/*K*_b_) is the dissociation constant, and *n* is the Hill coefficient.

### Preparation of DOX/^SS^BNPs

BSA (50 mg, 0.75 μmol) and DOX·HCl (2 mg, 3.45 μmol) were dissolved in H_2_O (2 mL) in a glass vial and stirred for 30 min. The pH of the solution was adjusted to 9.0 using NaOH. Then, EtOH (8 mL) was added at 1 mL min^–1^ to induce nanoaggregate formation. Then, DSP (1 mg, 2.47 μmol) was added, and the mixture was stirred for 24 h to form stable nanoparticles. The resultant nanoparticles were purified by centrifugation at 14,500 rpm for 30 min and resuspended in H_2_O for 3 cycles to eliminate free BSA, DOX, and excess cross-linking reagent. The collected nanoparticles were suspended in H_2_O (5 mL) and stored at 4 °C.

### Characterization of DOX/^SS^BNPs

The surface morphology of DOX/^SS^BNPs was determined by dropping the suspended solution on the copper grid for TEM. The hydrodynamic diameter and surface charge of DOX/^SS^BNPs were determined by DLS and zeta potential, respectively. The free amount of DOX was estimated by UV–vis absorption measurement. The DL and EE values were calculated as follows:

$$\mathrm{DL}=\frac{\mathrm{Amount}\;\mathrm{of}\;\mathrm{DOX}\;\mathrm{used}-\mathrm{Amount}\;\mathrm{of}\;\mathrm{free}\;\mathrm{DOX}}{\mathrm{Amount}\;\mathrm{of}\;\mathrm{nano}\mathrm{particles}}\times 100\%$$

$$\mathrm{EE}=\frac{\mathrm{Amount}\;\mathrm{of}\;\mathrm{DOX}\;\mathrm{used}-\mathrm{Amount}\;\mathrm{of}\;\mathrm{free}\;\mathrm{DOX}}{\mathrm{Amount}\;\mathrm{of}\;\mathrm{DOX}\;\mathrm{used}}\times 100\%$$

### TEM Studies

A diluted aqueous solution of DOX/^SS^BNPs (20 μL, 0.2 mg mL^–1^ of BNPs) or Ir-DOX/^SS^BNPs (20 μL, 5 μM of complex **1a** and 0.2 mg mL^–1^ of BNPs) treated with or without GSH (20 μL, 20 mM in H_2_O) was deposited onto a carbon-coated copper grid and the sample grid was left to dry at room temperature for 3 h before imaging. Bright-field TEM imaging was performed on a FEI Tecnai12 BioTWIN Transmission Electron Microscope operated at an acceleration voltage of 100 kV. All the TEM imaging was recorded by a 16-bit 2K × 2K FEI Eagle bottom mount camera.

### Cell Culture

HeLa cells were cultured in DMEM containing 10% FBS and 1% penicillin/streptomycin in an incubator at 37 °C under a 5% CO_2_ atmosphere. They were subcultured every 2–3 days.

### Cellular Uptake

HeLa cells were grown in a 35 mm tissue culture dish and incubated at 37 °C under a 5% CO_2_ atmosphere for 48 h. The growth medium was replaced by a medium containing the iridium­(III) complexes (5 μΜ) and incubated at 37 °C under a 5% CO_2_ atmosphere. After 2 h incubation, the medium was removed, and the cell layer was washed gently with PBS (1 mL × 3). The cells were trypsinized and harvested with PBS (2 mL). The resultant solution was heated with 65% HNO_3_ (2 mL) at 70 °C for 2 h, cooled to room temperature, and analyzed using a PerkinElmer NexION 2000 ICP-MS system.

### Live-Cell Confocal Imaging

HeLa cells in growth medium were seeded on a sterilized coverslip in a 35 mm tissue culture dish and grown at 37 °C under a 5% CO_2_ atmosphere for 48 h. In the costaining experiments, after treatment with complexes **1a**–**3a** or **1b**–**3b** (5 μM, 2 h, λ_ex_ = 405 nm, λ_em_ = 550–650 nm), the cells were washed with PBS (1 mL × 3) and further incubated with MitoTracker Deep Red (100 nM, 20 min, λ_ex_ = 635 nm, λ_em_ = 650–680 nm), ER-Tracker Green (1 μM, 20 min, λ_ex_ = 488 nm, λ_em_ = 500–550 nm), or LysoTracker Deep Red (100 nM, 30 min, λ_ex_ = 635 nm, λ_em_ = 650–680 nm) in growth medium at 37 °C under a 5% CO_2_ atmosphere. After washing with PBS (1 mL × 3), the cells were imaged using a Leica TCS SPE confocal microscope with an oil immersion 63× objective. In the nucleoli costaining experiments, after treatment with complex **2a** (5 μM, 2 h, λ_ex_ = 405 nm, λ_em_ = 550–650 nm), the cells were washed with PBS (1 mL × 3), fixed with paraformaldehyde (4%) in PBS for 15 min, permeabilized with Triton X-100 (0.1%) solution for 5 min, and blocked with BSA (3%) for 1 h. The cells were then incubated with fibrillarin antibody (2 μL mL^–1^) at 4 °C overnight. After incubation, the cells were thoroughly washed with PBS (1 mL × 3) and further incubated with Alexa Fluor 647-conjugated antirabbit IgG secondary antibody (2 μL mL^–1^, λ_ex_ = 635 nm, λ_em_ = 650–680 nm) in PBS at 4 °C for 1 h. After washing with PBS (1 mL × 3), the cells were imaged using a Leica TCS SPE confocal microscope with an oil immersion 63× objective.

For imaging of the intracellular delivery of Ir-DOX/^SS^BNPs, HeLa cells were incubated with **1a**-DOX/^SS^BNPs ([Ir] = 10 μM and [DOX/^SS^BNPs] = 0.2 mg mL^–1^) at 37 °C under a 5% CO_2_ atmosphere for 1, 2, and 4 h. After the treatment, the medium was removed, and the cells were washed with PBS (1 mL × 3) and then imaged using Leica TCS SPE confocal microscope with an oil immersion 63× objective. For the control experiment, HeLa cells were incubated with complex **1a** (10 μM) or DOX/^SS^BNPs (0.2 mg mL^–1^) at 37 °C under a 5% CO_2_ atmosphere for 4 h. After the treatment, the medium was removed, and the cells were washed with PBS (1 mL × 3) and then imaged using Leica TCS SPE confocal microscope with an oil immersion 63× objective. The iridium­(III) channel was excited at 405 nm with emission collected between 500–600 nm, while the DOX channel was excited at 488 nm with emission collected between 550–650 nm.

### MTT Assays

HeLa cells in growth medium were seeded in 96-well flat-bottomed microplates (ca. 10,000 cells per well) in a growth medium (100 μL) and incubated at 37 °C under a 5% CO_2_ atmosphere for 48 h. The growth medium was removed and replaced with the complexes, free DOX, DOX/^SS^BNPs, or Ir-DOX/^SS^BNPs in growth medium at 37 °C under a 5% CO_2_ atmosphere for 4 h. After the treatment, the medium was removed and replenished with phenol red-free growth medium (100 μL). One of the microplates was kept in the dark for 10 min, while the other microplate was irradiated with an LED (450 nm, 15.5 mW cm^–2^, 10 min) cellular photocytotoxicity irradiator (PURI Materials, Shenzhen, China). Then, the growth medium was replaced with fresh DMEM (100 μL) and the cells were further incubated at 37 °C under a 5% CO_2_ atmosphere for 20 h. After replenished with fresh DMEM (90 μL) and a solution of MTT in PBS (10 μL, 5 mg mL^–1^), the cells were incubated at 37 °C under a 5% CO_2_ atmosphere for 4 h. The growth medium was then removed and DMSO (100 μL) was added to each well. The microplates were further incubated at 37 °C under a 5% CO_2_ atmosphere for 15 min. The absorbance of the solutions at 570 nm was measured with a SPECTRAmax 340 microplate reader (Molecular Devices Corp., Sunnyvale, CA). The IC_50_ values of the complexes, free DOX, DOX/^SS^BNPs, and Ir-DOX/^SS^BNPs were determined from dose dependence of surviving cells.

### Annexin V–FITC/PI Double Staining Assay

HeLa cells in growth medium were seeded in 35 mm tissue culture dishes and incubated at 37 °C under a 5% CO_2_ atmosphere for 48 h. The growth medium was replaced by a medium containing **1a**-DOX/^SS^BNPs ([Ir] = 10 μM and [DOX/^SS^BNPs] = 0.2 mg mL^–1^) at 37 °C under a 5% CO_2_ atmosphere for 4 h. After the treatment, the medium was removed, and the cells were washed with PBS (1 mL × 3). For the control experiment, HeLa cells were incubated with complex **1a** (10 μM) or DOX/^SS^BNPs (0.2 mg mL^–1^) for 4 h, washed with PBS (1 mL × 3). After the treatment, the cells were irradiated at 450 nm (15.5 mW cm^–2^) for 10 min or kept in the dark. After incubation for 20 h, the medium was removed, and the cell layer was washed gently with PBS (1 mL × 3). The cell layer was then trypsinized and centrifuged at 1500 rpm for 1 min. The cell pellet was washed with PBS (1 mL) and subjected to centrifugation. The cells were resuspended in an Annexin V binding buffer (100 μL) in the flow cytometer tubes, followed by the addition of Alexa Fluor 647–Annexin V conjugate (5 μL) and PI (2 μL, 100 μg mL^–1^). The cell suspension was kept in the dark for 15 min. The suspension was then diluted with Annexin V binding buffer (400 μL) before analysis by a flow cytometer (Beckman CytoFLEX). The untreated cultured cells were used as a control group for background correction. The data were analyzed using the FlowJo V10 software.

## Supplementary Material



## Notes

Synthetic procedures, characterization data, electronic absorption data and spectra, emission spectra, DLS and zeta potential analyses, LSCM images, and cytotoxicity data (PDF)
