# Supplementary material for: Luminescent Cyclometalated Iridium(III) Polypyridine Complexes Modified with a Dendritic Guanidinium Moiety as Molecular Glues for Combined Chemo-Photodynamic Therapy
Source: Inorg Chem. 2025 Jul 17;64(29):15064–77. doi: 10.1021/acs.inorgchem.5c01970 (PMC12723684; doi:10.1021/acs.inorgchem.5c01970)
Supplement: Supplementary file 1 [file ic5c01970_si_001.pdf]

Supporting Information

Luminescent Cyclometalated Iridium(III) Polypyridine Complexes Modified with a Dendritic Guanidinium Moiety as Molecular Glues for Combined Chemo-Photodynamic Therapy

Jia-Hao Wang,[†] Lawrence Cho-Cheung Lee,[†] Alex Man-Hei Yip,[†,‡] Peter Kam-Keung Leung,[§] Kou Okuro,*[,||] and Kenneth Kam-Wing Lo*[,†,§]

[†]Department of Chemistry, City University of Hong Kong, Tat Chee Avenue, Kowloon, Hong Kong; Email: bhkenlo@cityu.edu.hk

[‡]Laboratory for Synthetic Chemistry and Chemical Biology Limited, Units 1503–1511, 15/F, Building 17W, Hong Kong Science Park, Pak Shek Kok, New Territories, Hong Kong

[§]State Key Laboratory of Terahertz and Millimeter Waves, City University of Hong Kong, Tat Chee Avenue, Kowloon, Hong Kong

[||]State Key Laboratory of Synthetic Chemistry and Department of Chemistry, The University of Hong Kong, Pokfulam Road, Pokfulam, Hong Kong; Email: okuro@hku.hk

Table of Contents

**Experimental**		**S5**
**Table S1**	Electronic absorption spectral data of the iridium(III) complexes at 298 K.	**S18**
**Table S2**	Zeta potential value, average diameter, and polydispersity index (PDI) value of ^{SS}BNPs, DOX/^{SS}BNPs, and Ir-DOX/^{SS}BNPs.	**S20**
**Figure S1**	Electronic absorption spectra of complexes **1a** – **3a** in H_2O (black) and MeOH (red) at 298 K.	**S21**
**Figure S2**	Electronic absorption spectra of complexes **1b** – **3b** in H_2O/MeOH (4:1, *v*/*v*) (black) and MeOH (red) at 298 K.	**S22**
**Figure S3**	Normalized emission spectra of complexes **1a** – **3a** in H_2O (black) and MeOH (red) at 298 K and alcohol glass at 77 K (blue).	**S23**
**Figure S4**	Normalized emission spectra of complexes **1b** – **3b** in H_2O/MeOH (4:1, *v*/*v*) (black) and MeOH (red) at 298 K and alcohol glass at 77 K (blue).	**S24**
**Figure S5**	LSCM images of HeLa cells incubated with (a) complex **2a** or (b) complex **3a** (5 μM, 2 h), and then MitoTracker Deep Red (100 nM, 20 min) at 37℃. Complexes **2a** and **3a**: λ_{ex} = 405 nm, λ_{em} = 550 – 650 nm. MitoTracker Deep Red: λ_{ex} = 635 nm, λ_{em} = 650 – 680 nm. Scale bar = 25 μm.	**S25**
**Figure S6**	LSCM images of HeLa cells incubated with (a) complex **1a** or (b) complex **2a** (5 μM, 2 h), and then ER-Tracker Green (100 nM, 20 min) at 37℃. Complexes **1a** and **2a**: λ_{ex} = 405 nm, λ_{em} = 550 – 650 nm. ER-Tracker Green: λ_{ex} = 488 nm, λ_{em} = 500 – 550 nm. Scale bar = 25 μm.	**S26**
**Figure S7**	LSCM images of HeLa cells incubated with (a) complex **1a**, (b) complex **2a**, or (c) complex **3a** (5 μM, 2 h), and then LysoTracker Deep Red (100 nM, 30 min) at 37℃. Complexes **1a** – **3a**: λ_{ex} = 405 nm, λ_{em} = 550 – 650 nm.	**S27**

	LysoTracker Deep Red: λ_{ex} = 635 nm, λ_{em} = 650 – 680 nm. Scale bar = 25 μm.	
**Figure S8**	Emission spectra of DOX in H_2O at 298 K upon irradiation at 480 nm.	**S28**
**Figure S9**	LSCM images of HeLa cells incubated with (a – c) DOX/^{SS}BNPs ([DOX] = 5 μg mL^{-1}) and (d – f) complex **1a** (5 μM) at 37℃ for 4 h. Ir channel: λ_{ex} = 405 nm, λ_{em} = 500 – 600 nm; DOX channel: λ_{ex} = 488 nm, λ_{em} = 550 – 650 nm. Scale bar = 25 μm.	**S29**
**Figure S10**	Viability of HeLa cells incubated with DOX/^{SS}BNPs (black) and DOX (red) at different concentrations at 37°C for 4 h, and subsequently incubated with blank medium for 20 h.	**S30**
**Figure S11**	Viability of HeLa cells incubated with complex **3a** at different concentrations at 37°C for 4 h, followed by incubation in the dark (blue) or irradiated at 450 nm (red) for 10 min (light dose = 15.5 mW cm^{-2}), and subsequently incubated with blank medium for 20 h.	**S31**
**Figure S12**	Flow cytometric analysis of HeLa cells treated with DOX/^{SS}BNPs ([DOX] = 5 μg mL^{-1}) or complex **1a** (5 μM) at 37°C for 4 h followed by incubation in the dark or irradiated at 450 nm for 10 min (light dose = 15.5 mW cm^{-2}), and subsequently incubated with blank medium for 20 h. They were then stained with Alexa Fluor 647–Annexin V conjugate (5 μL) and propidium iodide (PI) (2 μL, 100 μg mL^{-1}) and analyzed by flow cytometry using 488 and 638 nm excitation.	**S32**
**Figure S13**	ESI mass spectra of ligands (a) bpy-Ph-(TEG-N_3)$_3$ and (b) bpy-C4 in MeOH.	**S33**
**Figure S14**	ESI mass spectra of complexes **1** – **3** in MeOH.	**S34**
**Figure S15**	MALDI-TOF mass spectra of complexes **1a** – **3a**.	**S35**
**Figure S16**	ESI mass spectra of complexes **1b** – **3b** in MeOH.	**S36**

**Figure S17**	^{1}H NMR spectrum of bpy-Ph-(TEG-N_3)$_3$ in CD_3OD at 298 K.	**S37**
**Figure S18**	^{1}H NMR spectrum of bpy-C4 in CD_3OD at 298 K.	**S38**
**Figure S19**	^{1}H NMR spectrum of complex **1** in CD_3OD at 298 K.	**S39**
**Figure S20**	^{1}H NMR spectrum of complex **1a** in D_2O at 298 K.	**S40**
**Figure S21**	^{1}H NMR spectrum of complex **1b** in CD_3OD at 298 K.	**S41**
**Figure S22**	^{1}H NMR spectrum of complex **2** in CD_3OD at 298 K.	**S42**
**Figure S23**	^{1}H NMR spectrum of complex **2a** in D_2O at 298 K.	**S43**
**Figure S24**	^{1}H NMR spectrum of complex **2b** in CD_3OD at 298 K.	**S44**
**Figure S25**	^{1}H NMR spectrum of complex **3** in CD_3OD at 298 K.	**S45**
**Figure S26**	^{1}H NMR spectrum of complex **3a** in D_2O at 298 K.	**S46**
**Figure S27**	^{1}H NMR spectrum of complex **3b** in CD_3OD at 298 K.	**S47**
**References**		**S48**

EXPERIMENTAL

Synthesis and Characterization

4-(*N*-(3,4,5-Tris(2-(2-(2-azidoethoxy)ethoxy)ethoxy)phenylcarbonyl)aminomethyl)-4'-methyl-2,2'-bipyridine (bpy-Ph-(TEG-N_3)$_3$)

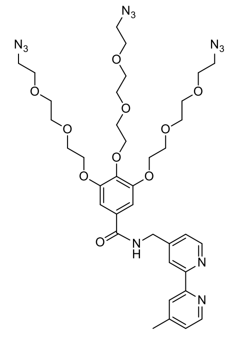

To a mixture of HOOC-Ph-(TEG-N_3)$_3$[1] (385 mg, 0.60 mmol) and *N*,*N*-diisopropylethylamine (DIPEA) (220 μL, 1.26 mmol) in CH_2Cl_2 (20 mL), 2-(1*H*-benzotriazole-1-yl)-1,1,3,3-tetramethyluronium hexafluorophosphate (HBTU) (274 mg, 0.72 mmol) was added, and the mixture was stirred at 298 K for 30 min. Then, 4-aminomethyl-4'-methyl-2,2'-bipyridine (bpy-CH_2-NH_2)[2] (100 mg, 0.5 mmol) was added, and the resulting mixture was stirred at 298 K for 18 h. Then, the mixture was diluted with CH_2Cl_2 (20 mL) and washed with H_2O (30 mL × 2) and brine (30 mL). The organic extract was dried over anhydrous $MgSO_4$, filtered, and evaporated to dryness under reduced pressure. The residual colorless oil was purified by column chromatography on silica gel using CH_2Cl_2/MeOH (15:1, *v*/*v*) as the eluent. The solvent was removed under reduced pressure to afford the product as a colorless oil. Yield: 257 mg (63%). ^{1}H NMR (300 MHz, CD_3OD, 298 K): δ 8.65 (d, J = 3.0 Hz, 1H, H6 of bpy), 8.53 (d, J = 3.0 Hz, 1H, H6' of bpy), 8.36 (s, 1H, H3 of bpy), 8.24 (s, 1H, H3' of bpy), 7.32 (d, J = 3.0 Hz, 1H, H5 of bpy), 7.18 – 7.14 (m, 3H, H5' of bpy, and H2 and H6 of phenyl ring), 6.88 (s, 1H, CONH), 4.73 (d, J = 3.0 Hz, 2H, CH_2 of bpy), 3.89 – 3.81 (m, 6H, PhOCH_2), 3.76 – 3.71 (m, 6H, PhOCH$_2$C*H*$_2$), 3.69 – 3.64 (m, 18H, OCH_2), 3.43 – 3.30 (m, 6H, CH_2N_3), 2.46 (s, 3H,

CH_3 of bpy). ESI-MS (positive-ion mode) *m/z* found: 823.6 $[M + H]^+$ and 845.6 $[M + Na]^+$ calcd: 822.88.

4-(*N*-(*n*-Propylcarbonyl)aminomethyl)-4'-methyl-2,2'-bipyridine (bpy-C4)

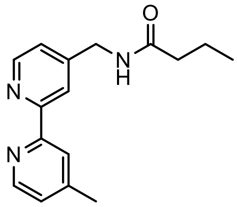

To a mixture of butyric acid (53 mg, 0.60 mmol) and DIPEA (220 μL, 1.26 mmol) in CH_2Cl_2 (20 mL), HBTU (274 mg, 0.72 mmol) was added, and the mixture was stirred at 298 K for 30 min. Then, bpy-CH_2-NH_2[2] (100 mg, 0.5 mmol) was added, and the resulting mixture was stirred at 298 K for 18 h. Then, the mixture was washed with H_2O (30 mL × 2) and brine (30 mL). The organic extract was dried over anhydrous $MgSO_4$, filtered, and evaporated to dryness under reduced pressure. The residual colorless oil was purified by column chromatography on silica gel using CH_2Cl_2/MeOH (20:1, *v*/*v*) as the eluent. The solvent was removed under reduced pressure to afford the product as a colorless oil. Yield: 103 mg (76%). ^1H NMR (300 MHz, CD_3OD, 298 K): δ 8.69 – 8.46 (m, 2H, H3 and H3' of bpy), 8.18 (d, J = 18.0 Hz, 2H, H6 and H6' of bpy), 7.43 – 7.27 (m, 2H, H5 and H5' of bpy), 4.52 (s, 2H, CH_2 of bpy), 2.49 (s, 3H, CH_3 of bpy), 2.31 (t, J = 6.0 Hz, 2H, $NHCOCH_2CH_2CH_3$ with *H*2 of $COC\mathit{H}_2$), 1.80 – 1.63 (m, 2H, $NHCOCH_2C\mathit{H}_2CH_3$), 1.00 (t, J = 6.0 Hz, 3H, $NHCOCH_2CH_2C\mathit{H}_3$). ESI-MS (positive-ion mode) *m*/*z* found: 270.4 $[M + H]^+$ and 292.4 $[M + Na]^+$ calcd: 269.35.

$[Ir(ppy)_2(bpy\text{-}Ph\text{-}(TEG\text{-}N_3)_3)](Cl)$ (**1**)

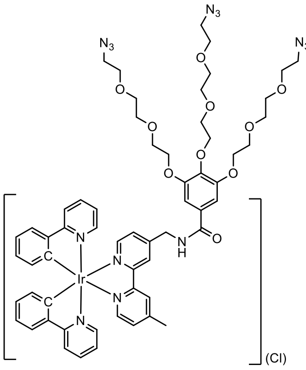

A mixture of $[Ir_2(ppy)_4Cl_2]$ (94 mg, 0.09 mmol) and bpy-Ph-$(TEG\text{-}N_3)_3$ (120 mg, 0.15 mmol) in CH_2Cl_2/MeOH (20 mL) (1:1, *v*/*v*) was stirred at 298 K under an inert atmosphere of N_2 in the dark for 18 h. The solvent was removed under reduced pressure and the residual red solid was purified by column chromatography on silica gel using CH_2Cl_2/MeOH (30:1, *v*/*v*) as the eluent. The solvent was removed under reduced pressure to afford the product as a yellow solid. Yield: 142.7 mg (72%). ^1H NMR (400 MHz, CD_3OD, 298 K): δ 9.15 (t, J = 6.0 Hz, 1H, NH of bpy-Ph-$(TEG\text{-}N_3)_3$), 8.68 (s, 1H, H3 of bpy), 8.58 (s, 1H, H3' of bpy), 8.18 – 8.08 (m, 2H, H3 of pyridyl ring of ppy), 7.96 (d, J = 6.0 Hz, 1H, H6 of pyridyl ring of ppy), 7.92 – 7.78 (m, 5H, H5, H6, and H6' of bpy and H3 of phenyl ring of ppy), 7.65 (t, J = 6.0 Hz, 2H, H4 of pyridyl ring of ppy), 7.50 (d, J = 6.0 Hz, 1H, H6' of pyridyl ring of ppy), 7.40 (d, J = 6.0 Hz, 1H, H5' of bpy), 7.27 (s, 2H, H2 and H6 of phenyl ring of bpy-Ph-$(TEG\text{-}N_3)_3$), 7.13 – 6.98 (m, 4H, H4 of phenyl ring and H5 of pyridyl ring of ppy), 6.95 – 6.85 (m, 2H, H5 and H5' of phenyl ring of ppy), 6.37 – 6.25 (m, 2H, H6 of phenyl ring of ppy), 4.76 (s, 2H, bpy-CH_2), 4.31 – 4.19 (m, 6H, $PhOCH_2$), 3.94 – 3.81 (m, 6H, $PhOCH_2CH_2$), 3.76– 3.63 (m, 18H, OCH_2), 3.38 – 3.34 (m, 6H, CH_2N_3), 2.59 (s, 3H, CH_3 on bpy). ESI-MS (positive-ion mode) *m*/*z* found: 1324.3 $[M – Cl^-]^+$ calcd: 1323.48.

[Ir(ppy)$_2$(bpy-Gu$_9$)](Cl)$_{10}$ (**1a**)

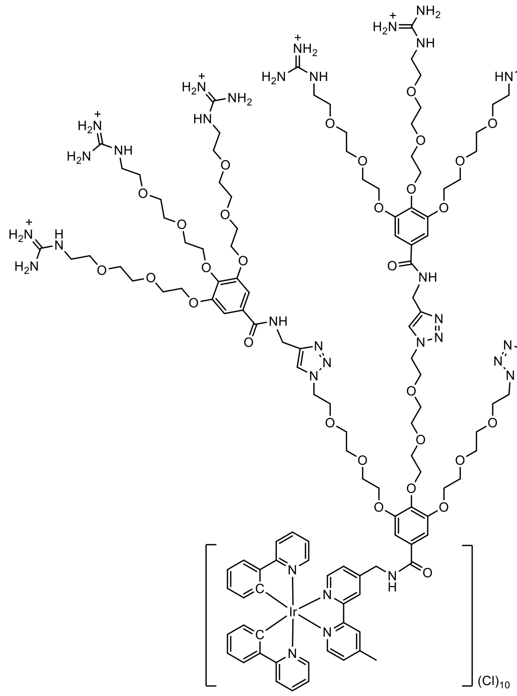

A mixture of alkyne-Ph-(TEG-GuBoc$_2$)$_3$[3] (49 mg, 36.7 μmol), complex **1** (11 mg, 8.16 μmol), $CuSO_4{\cdot}5H_2O$ (1 mg, 3.67 μmol), and sodium ascorbate (15 mg, 73.4 μmol) in THF/water (5 mL) (1/1, *v*/*v*) was stirred at 298 K for 12 h. Then, the mixture was diluted with ethyl acetate (10 mL) and washed with brine (20 mL). The organic extract was dried over anhydrous $MgSO_4$, filtered, and evaporated to dryness under reduced pressure. The red residue was purified by preparative thin layer chromatography (TLC) using CH_2Cl_2/MeOH (30:1, *v*/*v*) as the eluent. The solvent was removed under reduced pressure to afford the product as a yellow solid. A 1,4-dioxane solution of HCl (4 M) (5 mL) was added to the product, and the mixture was stirred at 298 K for 8 h. The reaction mixture was filtered, and the solid was washed with hexane to afford complex **1a** as a yellow solid. Yield: 14 mg (45%). ^{1}H NMR (400 MHz, D_2O, 298 K): δ 8.45 (s, 1H, H3 of bpy), 8.27 (s, 1H, H3' of bpy), 7.86 – 7.29 (m, 14H, H5 and H6 of bpy, H3, H4, H5, and H6 of pyridyl ring of ppy and H3 and H4 of phenyl ring of ppy), 7.17 – 7.08 (m, 1H, H6' of bpy), 6.97 – 6.55 (m, 14H, H5 of phenyl ring of ppy, H5' of bpy, H5 of triazole, and H2 and H6 of phenyl ring of bpy-Gu$_9$), 6.14 – 5.92 (m, 2H, H6 of phenyl ring of

ppy), 4.60 (s, 2H, CH_2 on bpy), 4.48 – 4.19 (m, 12H, CONHCH_2-triazole and OCH_2CH_2-triazole), 4.10 – 3.73 (m, 24H, $PhOCH_2$), 3.68 – 3.32 (m, 96H, OCH_2), 3.21 – 3.13 (m, 18H, CH_2NHCN), 2.34 (s, 3H, CH_3 on bpy). ^{13}C NMR (150 MHz, D_2O, 298 K): δ 20.7, 29.7, 34.9, 41.3, 43.4, 50.2, 54.0, 66.6, 68.2, 68.6, 68.8, 69.0, 69.1, 69.5, 69.7, 69.8, 70.1, 72.1, 106.3, 119.7, 122.4, 123.3, 124.6, 128.3, 129.1, 130.4, 131.3, 138.4, 139.7, 140.0, 144.0, 148.7, 150.7, 151.8, 154.7, 156.0, 157.2, 167.0, 168.4, 195.6. IR (KBr) $\tilde{\nu}$/cm^{-1}: 3414 (N–H), 1651 (C=O). MALDI-TOF-MS *m/z* found: 3504.4 [M – 9HCl – Cl$^-$]$^+$ calcd: 3503.98.

[Ir(ppy)$_2$(bpy-C4)](Cl) (**1b**)

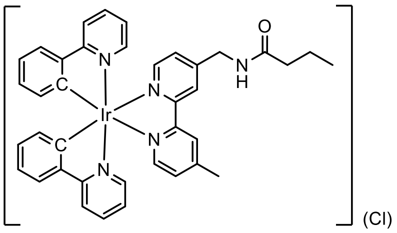

A mixture of [Ir$_2$(ppy)$_4$Cl$_2$] (64.3 mg, 0.06 mmol) and bpy-C4 (32.3 mg, 0.12 mmol) in CH_2Cl_2/MeOH (20 mL) (1:1, *v/v*) was stirred under an inert atmosphere of N_2 in the dark at 298 K for 18 h. The solvent was removed under reduced pressure and the residual red solid was purified by silica gel chromatography using CH_2Cl_2/MeOH (20:1, *v/v*) as the eluent. The solvent was removed under reduced pressure to afford the product as a yellow solid. Yield: 95 mg (80%). ^{1}H NMR (300 MHz, CD_3OD, 298 K): δ 8.58 (d, J = 6.0 Hz, 2H, H3 and H3' of bpy), 8.14 (d, J = 6.0 Hz, 2H, H3 of pyridyl ring of ppy), 7.95 (d, J = 6.0 Hz, 1H, H5' of bpy), 7.92 – 7.81 (m, 5H, H5, H6, and H6' of bpy and H3 of phenyl ring of ppy), 7.64 (d, J = 6.0 Hz, 2H, H4 of pyridyl ring of ppy), 7.42 (t, J = 6.0 Hz, 2H, H4 of phenyl ring of ppy), 7.12 – 6.98 (m, 4H, H5 and H6 of pyridyl ring of ppy), 6.90 (t, J = 6.0 Hz, 2H, H5 of phenyl ring of ppy), 6.31 (d, J = 6.0 Hz, 2H, H6 of phenyl ring of ppy), 4.57 (s, 2H, CH_2 on bpy), 2.61 (s, 3H, CH_3 on bpy), 2.31 (t, J = 6.0 Hz, 2H, $COC\mathit{H}_2CH_2CH_3$), 1.77 – 1.59 (m, 2H, $COCH_2C\mathit{H}_2CH_3$), 0.97 (t, J = 6.0 Hz, 3H, $COCH_2CH_2C\mathit{H}_3$). ^{13}C NMR (150 MHz, CD_3OD, 298 K): δ 175.3, 168.0, 157.1, 155.0, 152.6, 152.0, 150.3, 150.1, 149.6, 148.5, 143.8, 138.2, 131.4, 130.1, 128.8, 126.0, 125.1, 124.6, 123.0, 122.7, 122.2, 119.6, 41.8, 37.3, 29.4, 20.0, 18.8, 12.6. IR (KBr) $\tilde{\nu}$/cm^{-1}: 3435 (N–H), 1644 (C=O). ESI-MS (positive-ion mode) *m*/*z* found: 770.9 [M – Cl^-]$^+$ calcd: 770.25.

$[Ir(pq)_2(bpy\text{-}Ph\text{-}(TEG\text{-}N_3)_3)](Cl)$ (**2**)

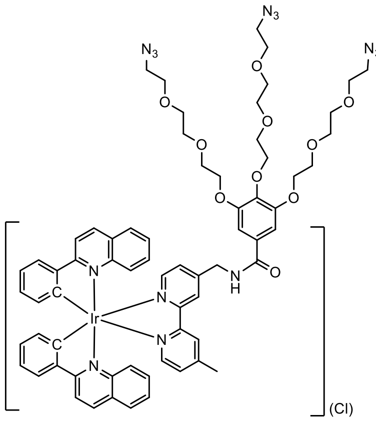

The synthetic procedure was similar to that of complex **1**, except that $[Ir_2(pq)_4Cl_2]$ (111.4 mg, 0.09 mmol) was used instead of $[Ir_2(ppy)_4Cl_2]$. Subsequent dryness of the solution under reduced pressure afforded complex **2** as a yellow solid. Yield: 160 mg (75%). ^{1}H NMR (400 MHz, CD_3OD, 298 K): δ 8.43 – 8.37 (m, 4H, H3 and H4 of quinolinyl ring of pq), 8.26 (s, 1H, H3 of bpy), 8.21 – 8.16 (m, 4H, H3 of phenyl ring of pq and H8 of quinolinyl ring of pq), 8.09 (d, J = 6.0 Hz, 1H, H3' of bpy), 7.83 (d, J = 9.0 Hz, 2H, H6 and H6' of bpy), 7.50 (d, J = 6.0 Hz, 1H, H5 of bpy), 7.45 – 7.34 (m, 5H, H5 and H7 of quinolinyl ring of pq and H5' of bpy), 7.21 (s, 2H, H2 and H6 of phenyl ring of bpy-Ph-$(TEG\text{-}N_3)_3$), 7.20 – 7.15 (m, 2H, H6 of quinolinyl ring of pq), 7.08 – 7.02 (m, 2H, H4 of phenyl ring of pq), 6.83 – 6.77 (t, J = 6.0 Hz, 2H, H5 of phenyl ring of pq), 6.53 – 6.48 (m, 2H, H6 of phenyl ring of pq), 4.65 (d, J = 12.0 Hz, 2H, bpy-CH_2), 4.29 – 4.18 (m, 6H, $PhOCH_2$), 3.92 – 3.89 (m, 4H, $PhOCH_2CH_2$), 3.86 – 3.82 (m, 2H, $PhOCH_2CH_2$), 3.78 – 3.55 (m, 18H, OCH_2), 3.37 – 3.34 (m, 6H, CH_2N_3), 2.46 (s, 3H, CH_3 on bpy). ESI-MS (positive-ion mode) m/z found: 1423.7 $[M - Cl^-]^+$ calcd: 1423.60.

[Ir(pq)$_2$(bpy-Gu$_9$)](Cl)$_{10}$ (**2a**)

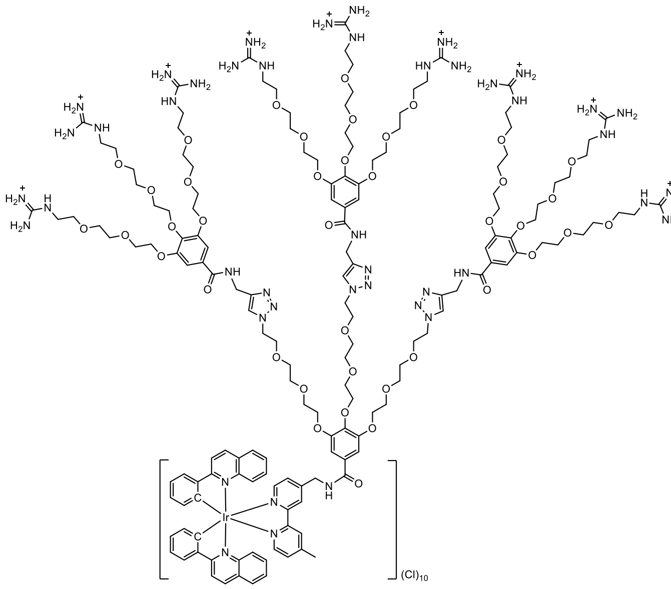

The synthetic procedure was similar to that of complex **1a**, except that complex **2** (15.1 mg, 10.3 μmol) was used instead of complex **1**. Subsequent dryness of the solution under reduced pressure afforded **2a** as a yellow solid. Yield: 15 mg (37%). ^{1}H NMR (300 MHz, D_2O, 298 K): δ 8.17 – 7.64 (m, 12H, H3 of phenyl ring of pq, H3, H5, and H7 of quinolinyl ring of pq, and H3, H3’, H5 and H5’ of bpy), 7.59 (d, J = 11.04 Hz, 1H, H6’ of bpy), 7.40 – 7.25 (m, 2H, H4 of quinolinyl ring of pq), 7.21 – 6.40 (m, 20H, H6 of bpy, H4, and H5 of phenyl ring of pq, H6, and H8 of quinolinyl ring of pq, H5 of triazole, and H2 and H6 of phenyl ring of bpy-Gu$_9$), 6.37 – 6.24 (t, J = 16 Hz, 2H, H6 of phenyl ring of pq,), 4.30 (br, 12H; $OCH_2C\mathit{H}_2$-triazole, triazole-$C\mathit{H}_2NH$), 4.14 (s, 2H, CH_2 on bpy), 4.01 – 3.76 (m, 26H, $PhOCH_2$, $CON\mathit{H}$), 3.64 – 3.35 (m, 106H, OCH_2, $CH_2N\mathit{H}CN$), 3.17 – 3.05 (m, 18H, $C\mathit{H}_2NHCN$), 2.18 (s, 3H, CH_3 on bpy). ^{13}C NMR (150 MHz, D_2O, 298 K): δ 20.3, 35.0, 41.3, 48.9, 50.1, 66.5, 68.1, 68.2, 68.5, 68.7, 69.0, 69.2, 69.5, 69.6, 69.7, 70.0, 72.1, 106.2, 122.7, 124.2, 126.6, 127.5, 129.2, 130.2, 133.9, 139.6, 140.5, 145.9, 147.0, 151.8, 157.1. IR (KBr) $\tilde{\nu}$/cm^{-1}: 3415 (N−H), 1638 (C=O). MALDI-TOF-MS (SA) *m/z* found: 3604.5 [M – 9HCl – Cl$^-$]$^+$ calcd: 3604.10.

[Ir(pq)$_2$(bpy-C4)](Cl) (**2b**)

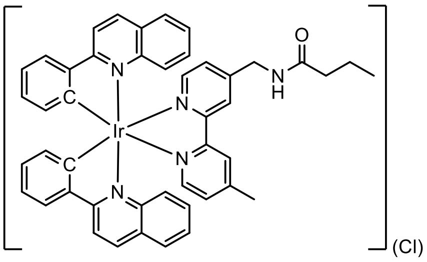

The synthetic procedure was similar to that of complex **1b**, except that [$Ir_2(pq)_4Cl_2$] (77 mg, 0.06 mmol) was used instead of [$Ir_2(ppy)_4Cl_2$]. Subsequent dryness of the solution under reduced pressure afforded complex **2b** as a yellow solid. Yield: 15 mg (81%). ^{1}H NMR (300 MHz, CD_3OD, 298 K): δ 8.47 – 8.36 (m, 4H, H3 and H4 of quinolinyl ring of pq), 8.25 – 8.06 (m, 6H, H3 and H3' of bpy, H3 of phenyl ring of pq, and H8 of quinolinyl ring of pq), 7.85 (d, J = 6.0 Hz, 2H, H6 and H6' of bpy), 7.48 – 7.30 (m, 6H, H5 and H7 of quinolinyl ring of pq and H5 and H5' of bpy), 7.18 (t, J = 6.0 Hz, 2H, H6 of quinolinyl ring of pq), 7.10 – 7.00 (m, 2H, H4 of phenyl ring of pq), 6.81 (t, J = 6.0 Hz, 2H, H5 of phenyl ring of pq), 6.51 (t, J = 6.0 Hz, 2H, H6 of phenyl ring of pq), 4.45 (d, J = 3.0, 2H, CH_2 on bpy), 2.46 (s, 3H, CH_3 on bpy), 2.24 (t, J = 6.0 Hz, 2H, $COCH_2CH_2CH_3$ with $C\mathit{H}_2$ first), 1.71 – 1.56 (m, 2H, $COCH_2C\mathit{H}_2CH_3$), 0.91 (t, J = 6.0 Hz, 3H, $COCH_2CH_2C\mathit{H}_3$). ^{13}C NMR (150 MHz, CD_3OD, 298 K): δ 175.1, 170.3, 155.8, 155.2, 152.7, 152.1, 151.0, 147.5, 147.4, 147.0, 145.8, 139.9, 134.2, 130.5, 130.3, 128.9, 128.5, 127.8, 127.0, 126.4, 125.7, 124.7, 124.4, 122.6, 122.1, 117.6, 41.4, 37.3, 29.3, 19.7, 18.8, 12.6. IR (KBr) $\tilde{\nu}$/cm^{-1}: 3447 (N–H), 1638 (C=O). ESI-MS (positive-ion mode) m/z found: 870.9 [M – Cl$^-$]$^+$ calcd: 870.28.

[Ir(bsn)$_2$(bpy-Ph-(TEG-N$_3$)$_3$)](Cl) (**3**)

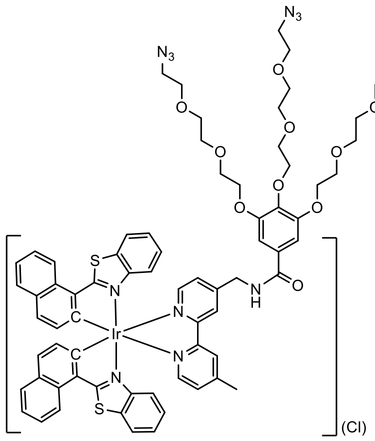

The synthetic procedure was similar to that of complex **1**, except that [Ir$_2$(bsn)$_4$Cl$_2$] (130 mg, 0.09 mmol) was used instead of [Ir$_2$(ppy)$_4$Cl$_2$]. Subsequent dryness of the solution under reduced pressure afforded complex **3** as a yellow solid. Yield: 149 mg (65%). ^{1}H NMR (300 MHz, CD$_3$OD, 298 K): δ 8.72 (dd, J = 8.4, 2.0 Hz, 2H, H8 of naphthyl ring of bsn), 8.64 (s, 1H, H3 of bpy), 8.55 (s, 1H, H3' of bpy), 8.16 (d, J = 8.0 Hz, 2H, H5 and H5' of naphthyl ring of bsn), 8.06 (d, J = 5.7 Hz, 1H, H6 of bpy), 7.95 (d, J = 5.7 Hz, 1H, H6' of bpy), 7.85 – 7.69 (m, 4H, H4 of benzothiazolyl ring of bsn and H7 of naphthyl ring of bsn), 7.57 – 7.41 (m, 6H, H5 and H6 of benzothiazolyl ring of bsn and H6 of naphthyl ring of bsn), 7.37 – 7.30 (m, 2H, H5 and H5' of bpy), 7.25 – 7.12 (m, 4H, H2 and H6 of phenyl ring of bpy-Ph-(TEG-N$_3$)$_3$ and H4 and H4' of naphthyl ring of bsn), 6.63 (dd, J = 8.4, 3.4 Hz, 2H, H7 of benzothiazolyl ring of bsn), 6.48 (d, J = 8.5 Hz, 1H, H3 of naphthyl ring of bsn), 6.38 (d, J = 8.4 Hz, 1H, H3 of naphthyl ring of bsn), 4.76 (d, J = 7.0 Hz, 2H, bpy-CH$_2$), 4.26 – 4.17 (m, 6H, PhOCH$_2$), 3.90 – 3.80 (m, 6H, PhOCH$_2$CH_2), 3.75 – 3.57 (m, 18H, OCH$_2$), 3.32 – 3.26 (m, 6H, CH$_2$N$_3$), 2.57 (s, 3H, CH$_3$ on bpy). ESI-MS (positive-ion mode) *m*/*z* found: 1535.7 [M – Cl$^-$]$^+$ calcd: 1535.77.

[Ir(bsn)$_2$(bpy-Gu$_9$)](Cl)$_{10}$ (**3a**)

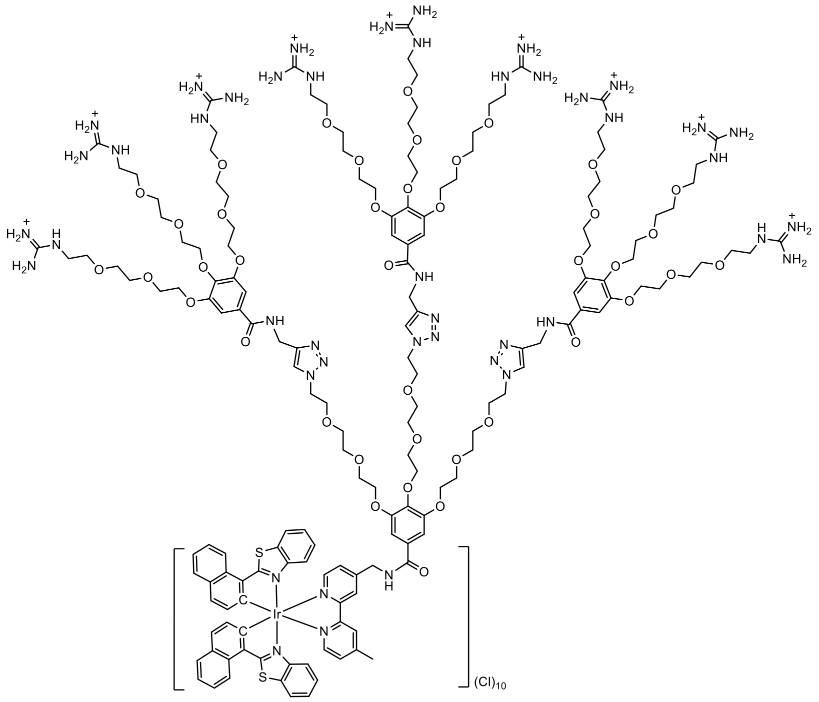

The synthetic procedure was similar to that of complex **1a**, except that complex **3** (21 mg, 13.3 μmol) was used instead of complex **1**. Subsequent dryness of the solution under reduced pressure afforded complex **3a** as a yellow solid. Yield: 20 mg (38%). ^{1}H NMR (400 MHz, D_2O, 298 K): δ 8.57 – 8.23 (m, 3H, H3 of bpy and H8 of naphthyl ring of bsn), 8.20 – 6.48 (m, 31H, H5, H6, H3', H5' and H6' of bpy, H4, H5, H6 and H7 of naphthyl ring of bsn, H4, H5, H6 and H7 of benzothiazolyl ring of bsn, H5 of triazole, and H2 and H6 of phenyl ring of bpy-Gu$_9$), 6.39 – 5.97 (m, 3H, H7 of benzothiazolyl ring and H3 of naphthyl ring of bsn), 4.40 – 4.14 (m, 8H, CH_2 on bpy, CONHCH_2-triazole), 4.06 – 2.86 (m, 144H, OCH_2, OCH_2CH_2-triazole, PhOCH_2, CH_2NHCN), 2.34 (s, 3H, CH_3 on bpy). ^{13}C NMR (150 MHz, D_2O, 298 K): δ 35.0, 41.2, 48.9, 50.3, 66.6, 68.2, 68.3, 68.8, 68.9, 69.0, 69.1, 69.4, 69.6, 69.7, 70.0, 72.1, 106.4, 120.0 – 135.0, 139.8, 144.0, 151.8, 157.1. IR (KBr) $\tilde{\nu}$/cm^{-1}: 3416 (N–H), 1638 (C=O). MALDI-TOF-MS (SA) *m/z* found: 3716.4 [M – 9HCl – Cl$^-$]$^+$ calcd: 3716.27.

[Ir(bsn)$_2$(bpy-C4)](Cl) (**3b**)

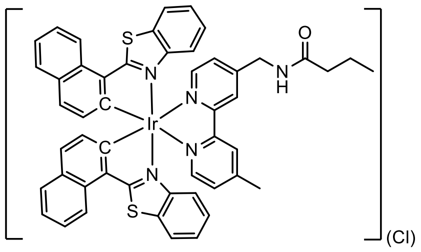

The synthetic procedure was similar to that of complex **1b**, except that [Ir$_2$(bsn)$_4$Cl$_2$] (90 mg, 0.06 mmol) was used instead of [Ir$_2$(ppy)$_4$Cl$_2$]. Subsequent dryness of the solution under reduced pressure afforded complex **3b** as a yellow solid. Yield: 15 mg (81%). ^{1}H NMR (300 MHz, CD$_3$OD, 298 K): δ 8.73 (d, J = 9.0 Hz, 2H, H8 of naphthyl ring of bsn), 8.55 (d, J = 9.0 Hz, 2H, H3 and H3' of bpy), 8.18 (d, J = 9.0 Hz, 2H, H5 of naphthyl ring of bsn), 8.04 (d, J = 6.0 Hz, 1H, H6 of bpy), 7.95 (d, J = 6.0 Hz, 1H, H6' of bpy), 7.87 – 7.70 (m, 4H, H4 of benzothiazolyl ring and H7 of naphthyl ring of bsn), 7.57 – 7.39 (m, 6H, H5 and H6 of benzothiazolyl ring and H6 of naphthyl ring of bsn), 7.35 (d, J = 9.0 Hz, 2H, H5 and H5' of bpy), 7.18 (t, J = 6.0 Hz, 2H, H4 of naphthyl ring of bsn), 6.64 (dd, J = 9.0, 6.0 Hz, 2H, H7 of benzothiazolyl ring of bsn), 6.47 (d, J = 9.0 Hz, 1H, H3 of naphthyl ring of bsn), 6.38 (d, J = 9.0 Hz, 1H, H3 of naphthyl ring of bsn), 4.65 – 4.47 (m, 2H, CH$_2$ on bpy), 2.57 (s, 3H, CH$_3$ on bpy), 2.26 (t, J = 6.0 Hz, 2H, COCH_2CH$_2$CH$_3$), 1.71 – 1.56 (m, 2H, COCH$_2$CH_2CH$_3$), 0.91 (t, J = 6.0 Hz, 3H, COCH$_2$CH$_2$CH_3). ^{13}C NMR (150 MHz, CD$_3$OD, 298 K): δ 178.9, 178.8, 175.3, 156.5, 155.9, 153.7, 153.1, 149.8, 149.3, 147.8, 133.9, 132.2, 131.7, 132.2, 131.1, 130.9, 130.1, 129.2, 128.3, 127.9, 127.8, 126.4, 125.5, 125.1, 124.1, 123.3, 122.7, 121.3, 117.0, 116.9, 41.7, 37.3, 29.3, 20.0, 18.8, 12.6. IR (KBr) $\tilde{\nu}$/cm^{-1}: 3447 (N–H), 1638 (C=O). ESI-MS (positive-ion mode) m/z found: 982.9 [M – Cl$^-$]$^+$ calcd: 982.23.

Table S1. Electronic absorption spectral data of the iridium(III) complexes at 298 K.

Complex	Solvent	λ_{abs}/nm (ε/dm^{3}mol^{-1}cm^{-1})
**1a**	MeOH	260 (78,710), 276 (59,400), 312 sh (20,080), 340 sh (8,615), 383 sh (5,235), 418 (2,940)
	H_2O	260 (77,910), 276 (58,540), 312 sh (20,680), 340 sh (8,525), 383 sh (4,925), 418 (2,620)
**1b**	MeOH	260 (37,200), 276 (30,345), 312 sh (14,990), 340 sh (7,050), 383 sh (4,290), 418 (2,370)
	H_2O^a	260 (38,685), 276 (30,725), 312 sh (15,230), 340 sh (7,400), 383 sh (4,390), 418 (2,390)
**2a**	MeOH	261 (76,360), 285 sh (52,460), 308 sh (26,885), 336 (20,195), 351 sh (17,920), 442 (4,660)
	H_2O	261 (74,275), 285 sh (50,805), 308 sh (27,270), 336 (20,145), 351 sh (17,650), 442 (4,630)
**2b**	MeOH	261 (40,605), 285 sh (36,810), 308 sh (18,920), 336 (18,830), 351 sh (16,420), 442 (4,095)
	H_2O^a	261 (40,165), 285 sh (34,190), 308 sh (18,435), 336 (18,005), 351 sh (15,420), 442 (3,930)
**3a**	MeOH	262 (79,915), 285 sh (51,115), 347 (27,895), 381 sh (15,610), 404 (11,660), 441 (8,080), 475 (7,755)
	H_2O	262 (74,135), 285 sh (50,000), 347 (25,945), 381 sh (15,095), 404 (11,115), 441 (7,575), 475 (7,500)
**3b**	MeOH	262 (37,520), 285 sh (29,750), 347 (22,835), 381 sh (12,550), 404 (9,385), 441 (6,630), 475 (6,330)

H_2O[a]	262 (39,335), 285 sh (32,130), 347 (23,650), 381 sh (13,400), 404 (10,130), 441 (7,110), 475 (6,705)

[a] H_2O/MeOH (4:1, *v*/*v*).

Table S2. Zeta potential value, average diameter, and polydispersity index (PDI) value of ^{SS}BNPs, DOX/^{SS}BNPs, and Ir-DOX/^{SS}BNPs.

Nanoparticle	Zeta potential/mV	Average diameter/nm	PDI
^{SS}BNPs	–28.6	143.1	0.096
DOX/^{SS}BNPs	–20.2	179.7	0.112
Ir-DOX/^{SS}BNPs	+19	194.2	0.121

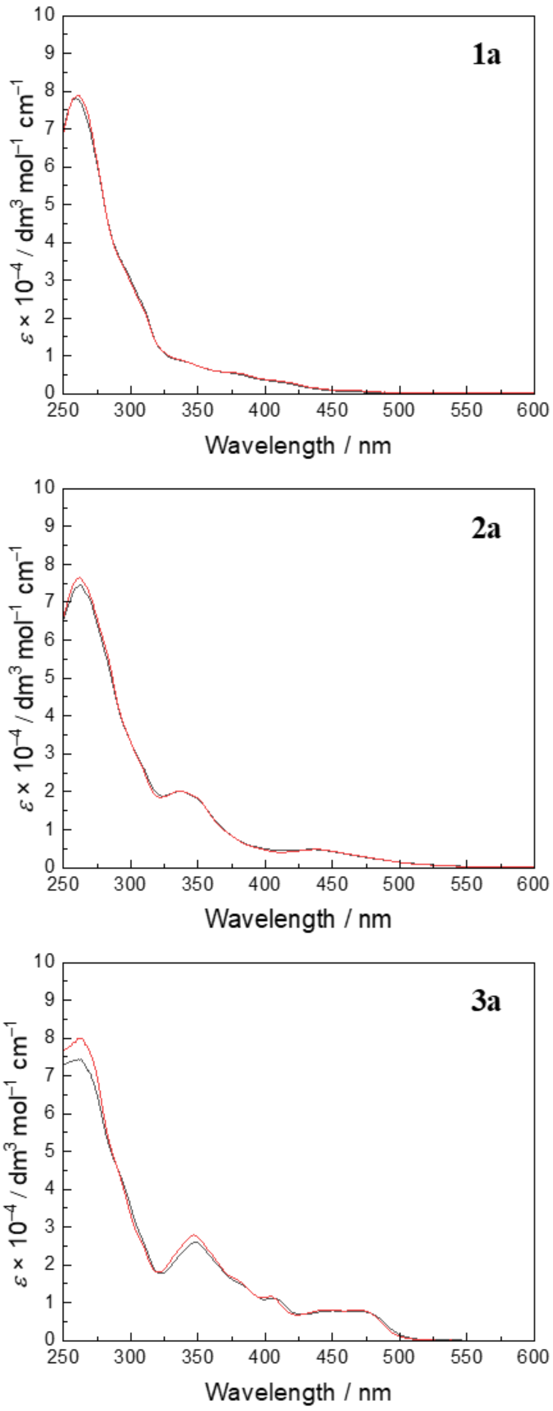

Figure S1. Electronic absorption spectra of complexes **1a** – **3a** in H_2O (black) and MeOH (red) at 298 K.

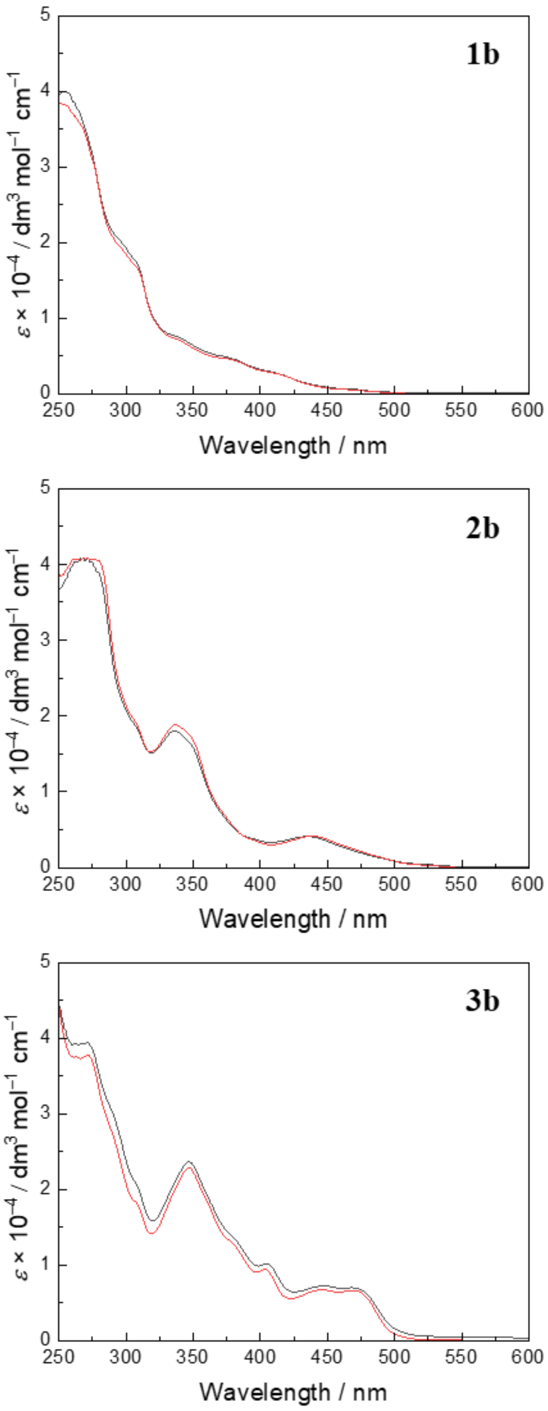

Figure S2. Electronic absorption spectra of complexes **1b** – **3b** in H_2O/MeOH (4:1, *v*/*v*) (black) and MeOH (red) at 298 K.

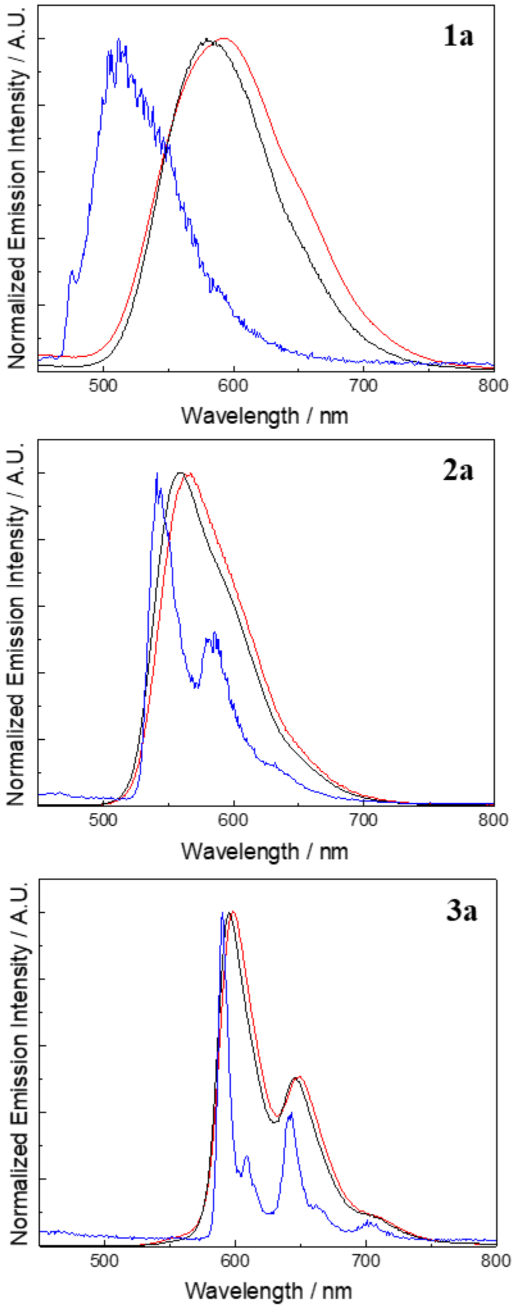

Figure S3. Normalized emission spectra of complexes **1a** – **3a** in H_2O (black) and MeOH (red) at 298 K and alcohol glass at 77 K (blue).

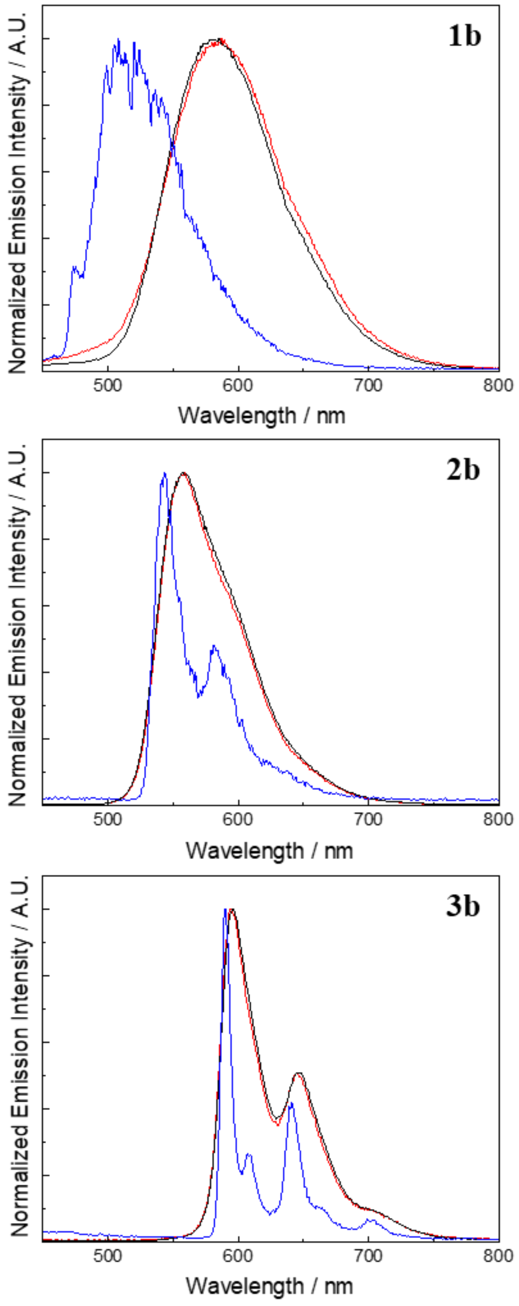

Figure S4. Normalized emission spectra of complexes **1b** – **3b** in H_2O/MeOH (4:1, *v*/*v*) (black) and MeOH (red) at 298 K and alcohol glass at 77 K (blue).

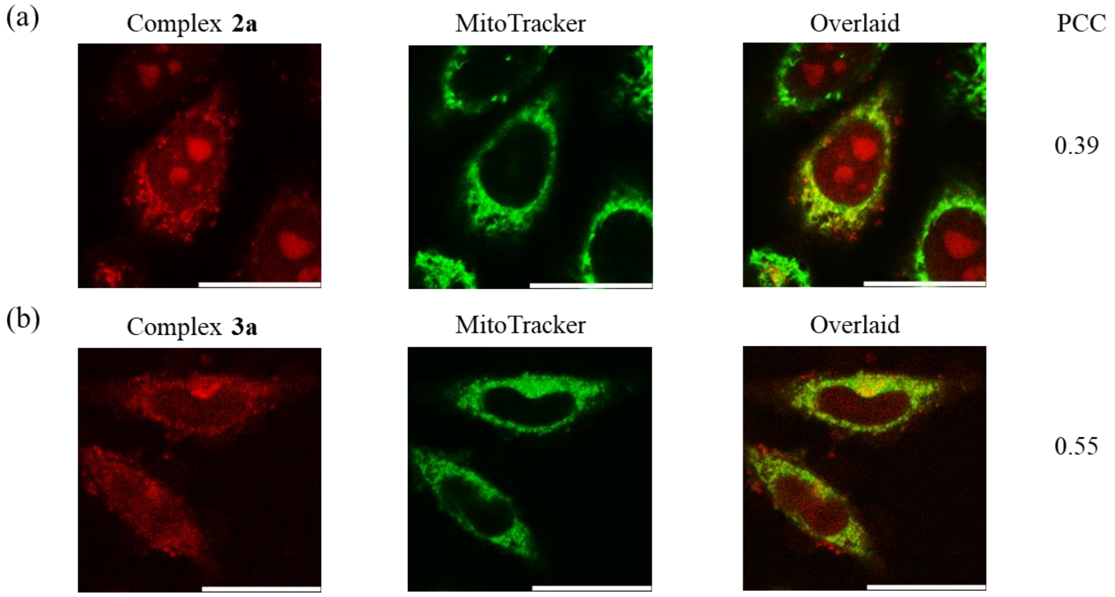

Figure S5. LSCM images of HeLa cells incubated with (a) complex **2a** or (b) complex **3a** (5 μM, 2 h), and then MitoTracker Deep Red (100 nM, 20 min) at 37°C. Complexes **2a** and **3a**: λ_{ex} = 405 nm, λ_{em} = 550 – 650 nm. MitoTracker Deep Red: λ_{ex} = 635 nm, λ_{em} = 650 – 680 nm. Scale bar = 25 μm.

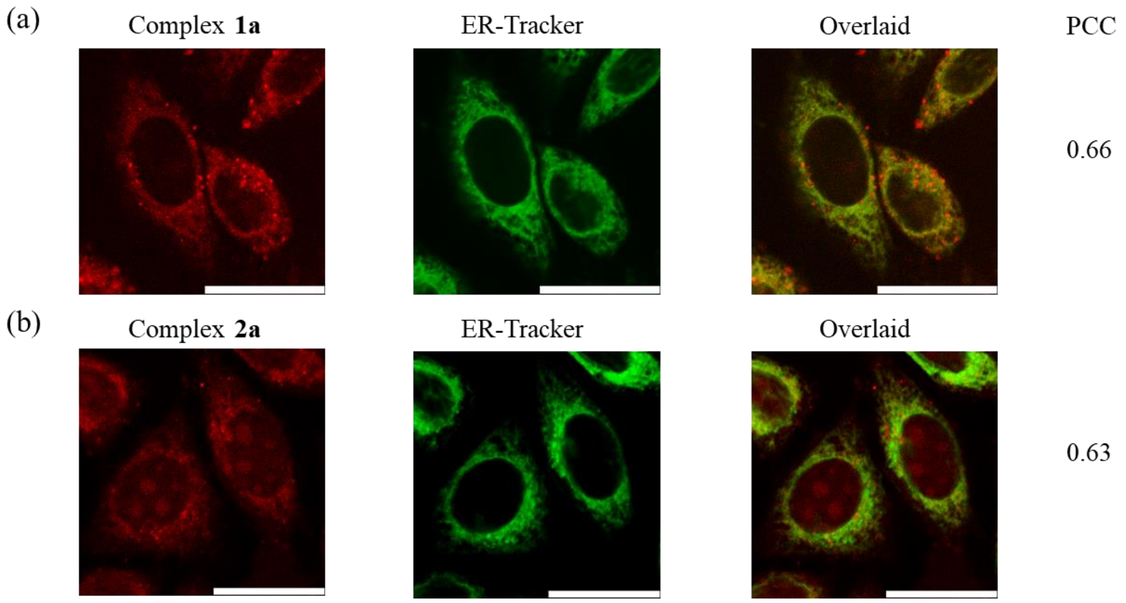

Figure S6. LSCM images of HeLa cells incubated with (a) complex **1a** or (b) complex **2a** (5 μM, 2 h), and then ER-Tracker Green (100 nM, 20 min) at 37°C. Complexes **1a** and **2a**: λ_{ex} = 405 nm, λ_{em} = 550 – 650 nm. ER-Tracker Green: λ_{ex} = 488 nm, λ_{em} = 500 – 550 nm. Scale bar = 25 μm.

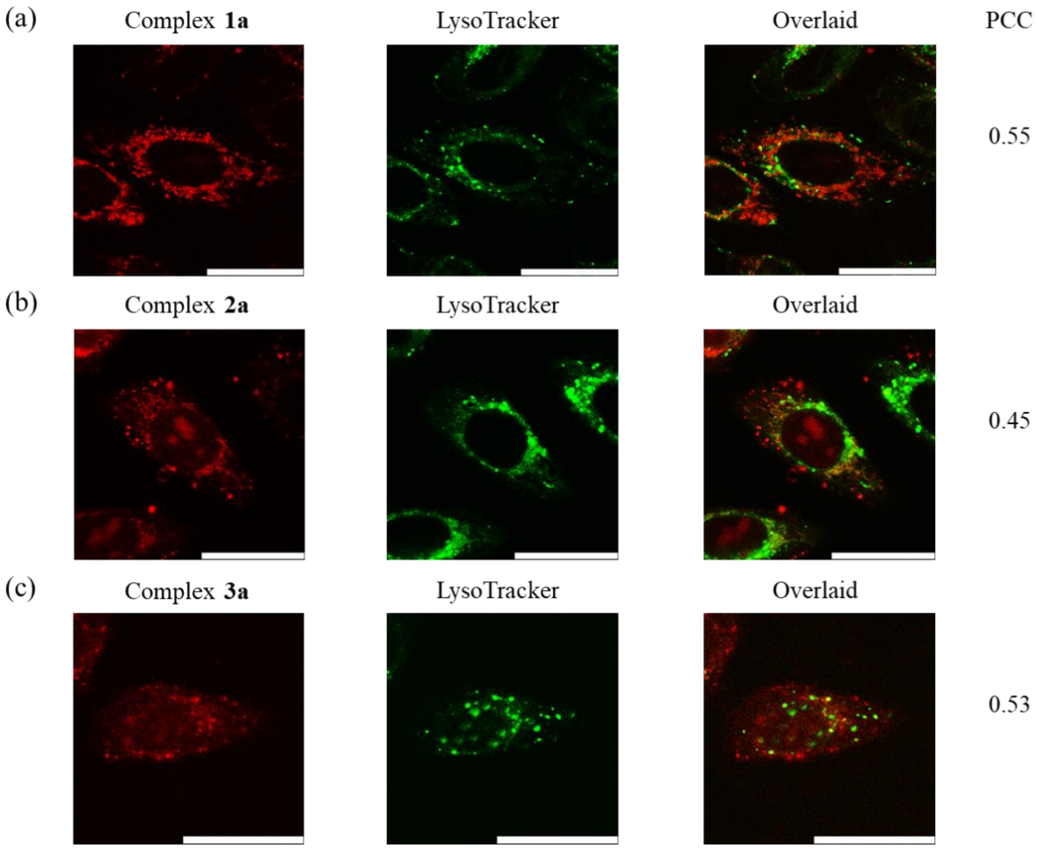

Figure S7. LSCM images of HeLa cells incubated with (a) complex **1a**, (b) complex **2a**, or (c) complex **3a** (5 μM, 2 h), and then LysoTracker Deep Red (100 nM, 30 min) at 37°C. Complexes **1a** – **3a**: λ_{ex} = 405 nm, λ_{em} = 550 – 650 nm. LysoTracker Deep Red: λ_{ex} = 635 nm, λ_{em} = 650 – 680 nm. Scale bar = 25 μm.

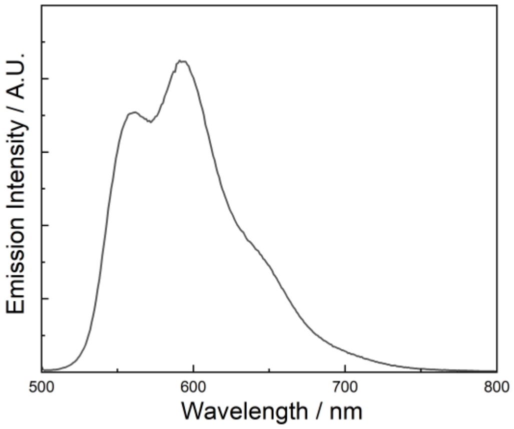

Figure S8. Emission spectra of DOX in H_2O at 298 K upon irradiation at 480 nm.

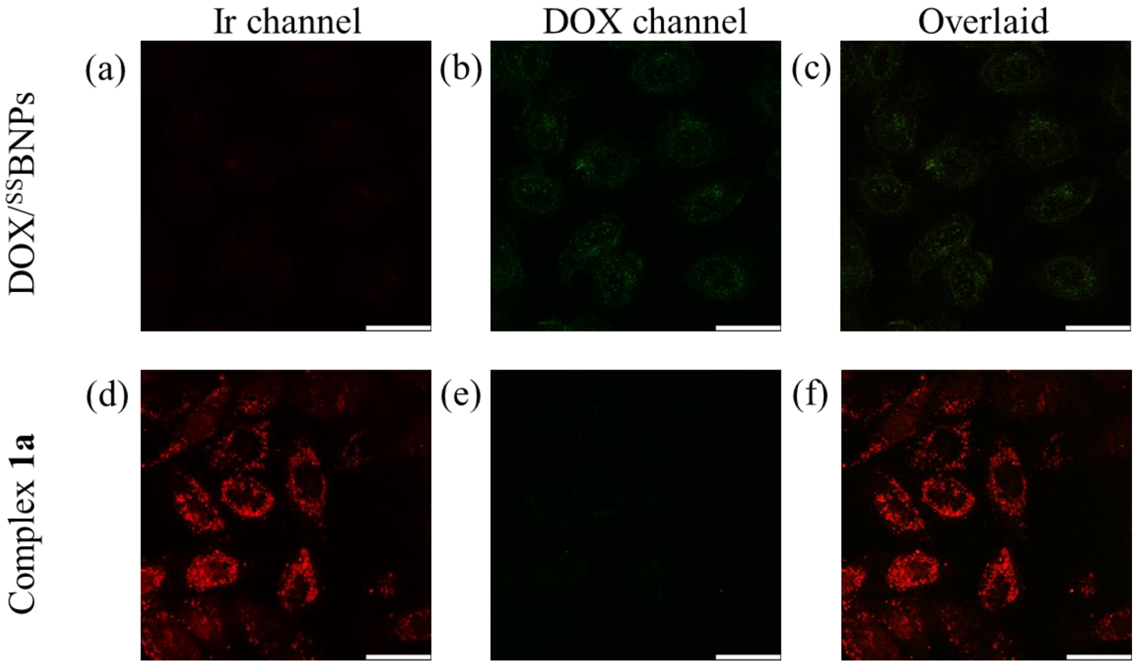

Figure S9. LSCM images of HeLa cells incubated with (a – c) DOX/^{SS}BNPs ([DOX] = 5 μg mL^{-1}) and (d – f) complex **1a** (5 μM) at 37°C for 4 h. Ir channel: λ_{ex} = 405 nm, λ_{em} = 500 – 600 nm; DOX channel: λ_{ex} = 488 nm, λ_{em} = 550 – 650 nm. Scale bar = 25 μm.

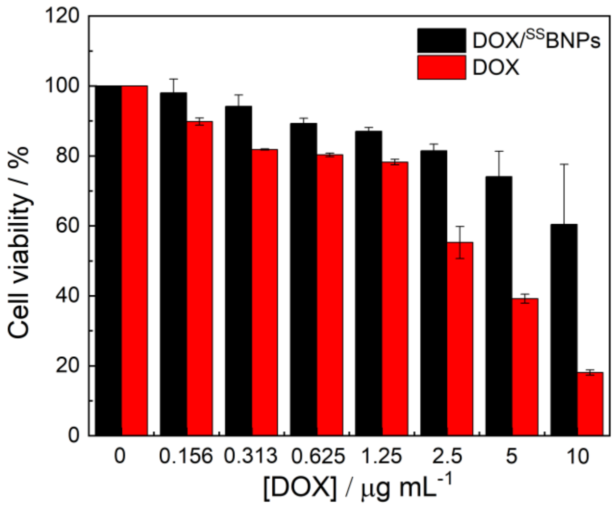

Figure S10. Viability of HeLa cells incubated with DOX/^{SS}BNPs (black) and DOX (red) at different concentrations at 37°C for 4 h, and subsequently incubated with blank medium for 20 h.

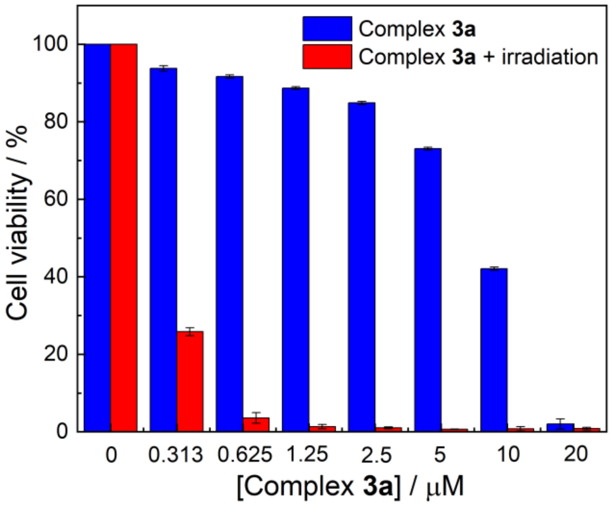

Figure S11. Viability of HeLa cells incubated with complex **3a** at different concentrations at 37°C for 4 h, followed by incubation in the dark (blue) or irradiated at 450 nm (red) for 10 min (light dose = 15.5 mW cm^{-2}), and subsequently incubated with blank medium for 20 h.

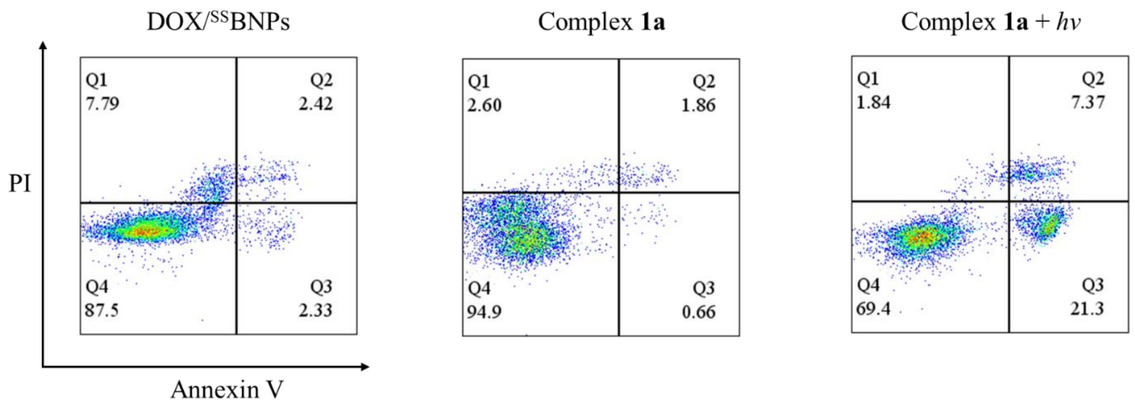

Figure S12. Flow cytometric analysis of HeLa cells treated with DOX/^{SS}BNPs ([DOX] = 5 μg mL^{-1}) or complex **1a** (5 μM) at 37°C for 4 h followed by incubation in the dark or irradiated at 450 nm for 10 min (light dose = 15.5 mW cm^{-2}), and subsequently incubated with blank medium for 20 h. They were then stained with Alexa Fluor 647–Annexin V conjugate (5 μL) and propidium iodide (PI) (2 μL, 100 μg mL^{-1}) and analyzed by flow cytometry using 488 and 638 nm excitation.

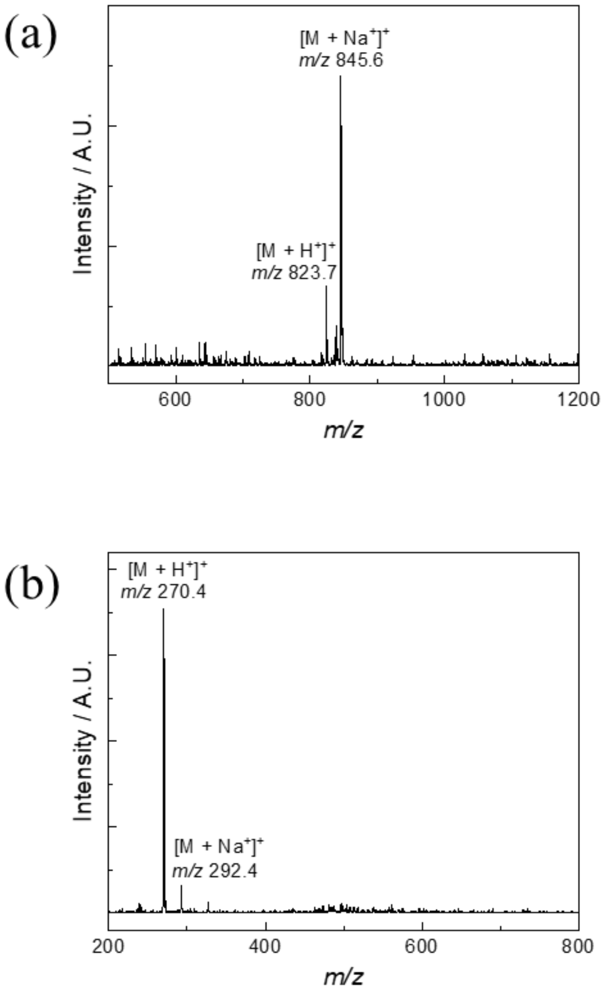

Figure S13. ESI mass spectra of ligands (a) bpy-Ph-(TEG-N_3)$_3$ and (b) bpy-C4 in MeOH.

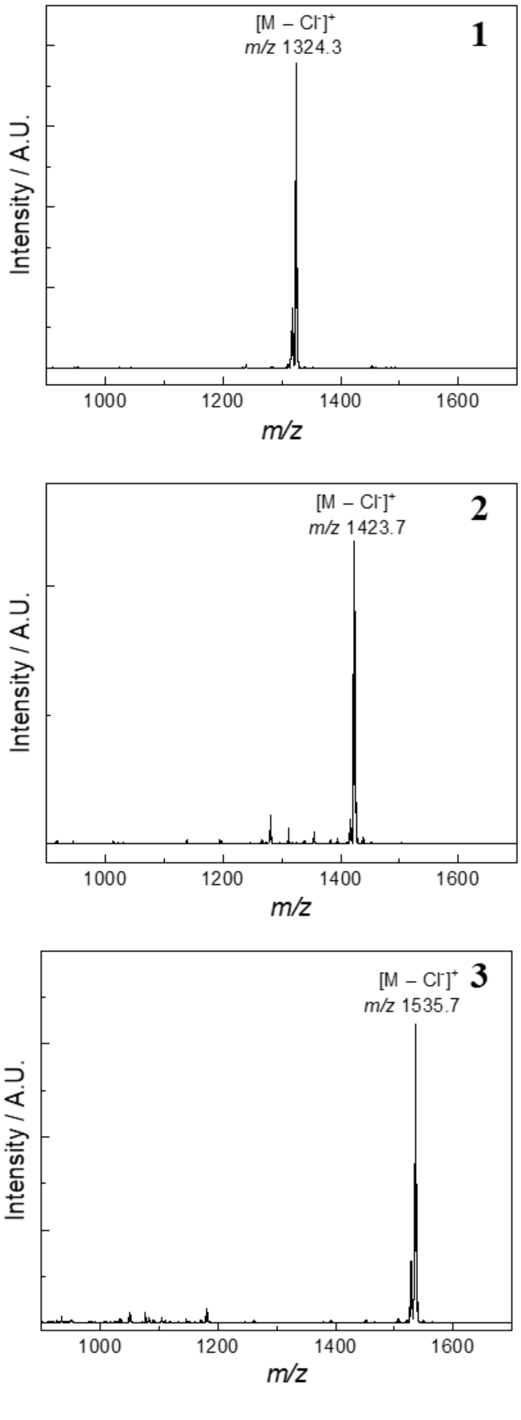

Figure S14. ESI mass spectra of complexes **1** – **3** in MeOH.

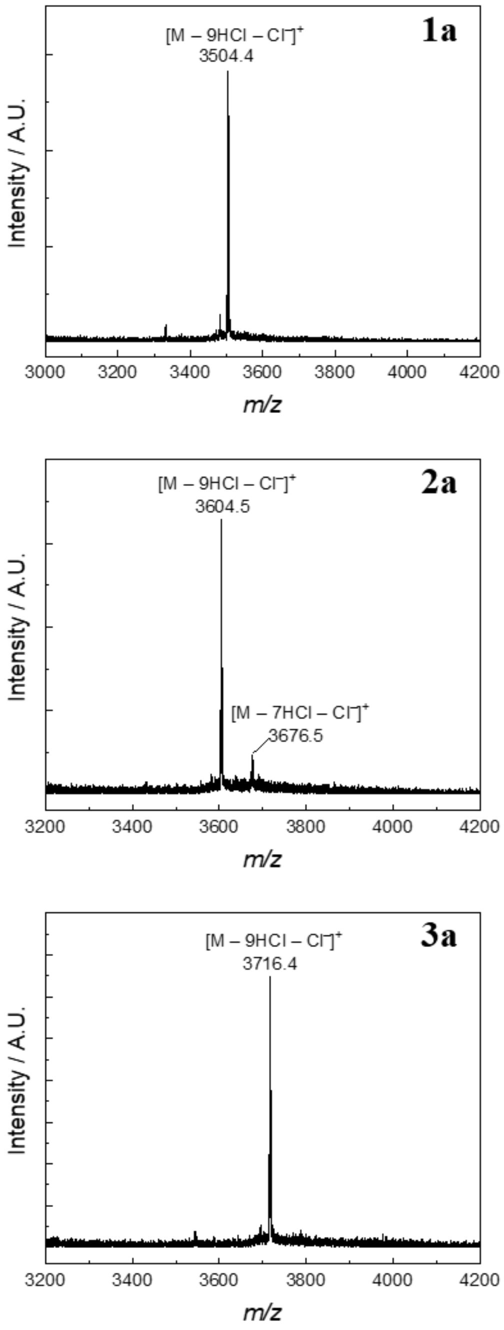

Figure S15. MALDI-TOF mass spectra of complexes **1a** – **3a**.

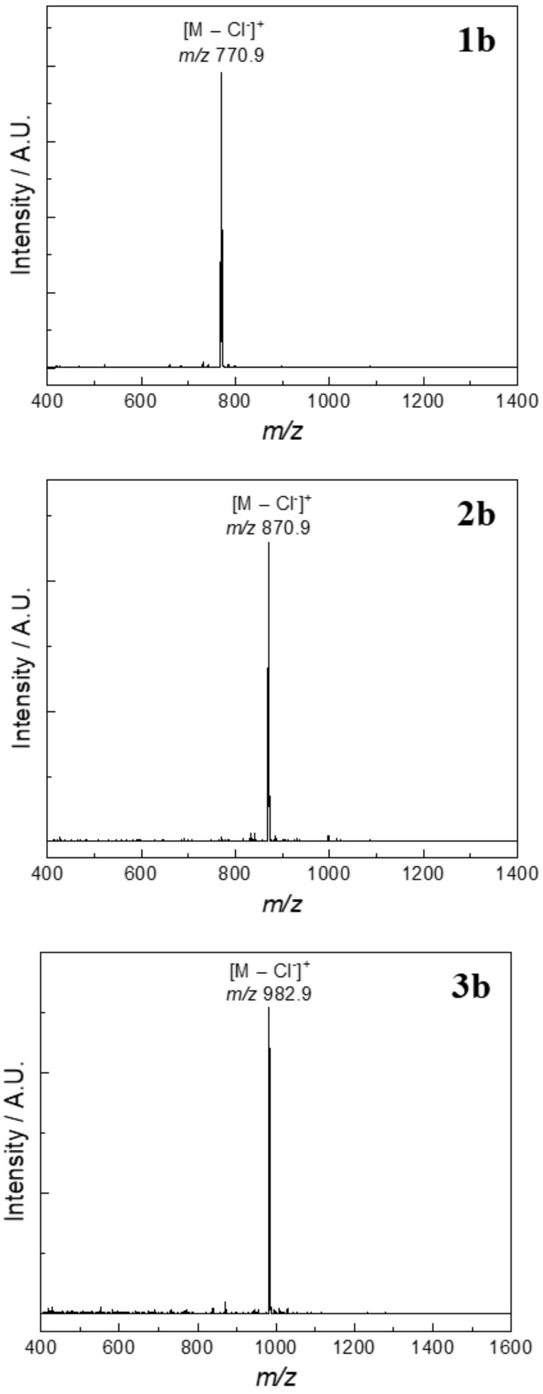

Figure S16. ESI mass spectra of complexes **1b** – **3b** in MeOH.

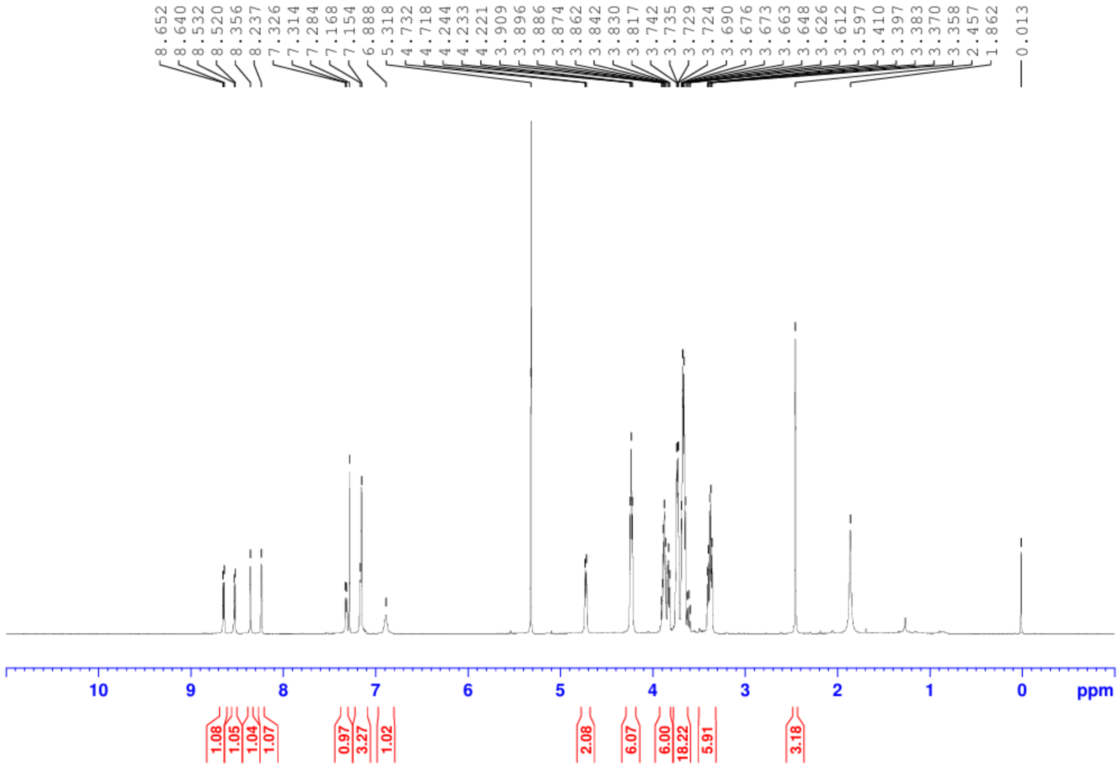

Figure S17. ^{1}H NMR spectrum of bpy-Ph-(TEG-N_3)$_3$ in CD_3OD at 298 K.

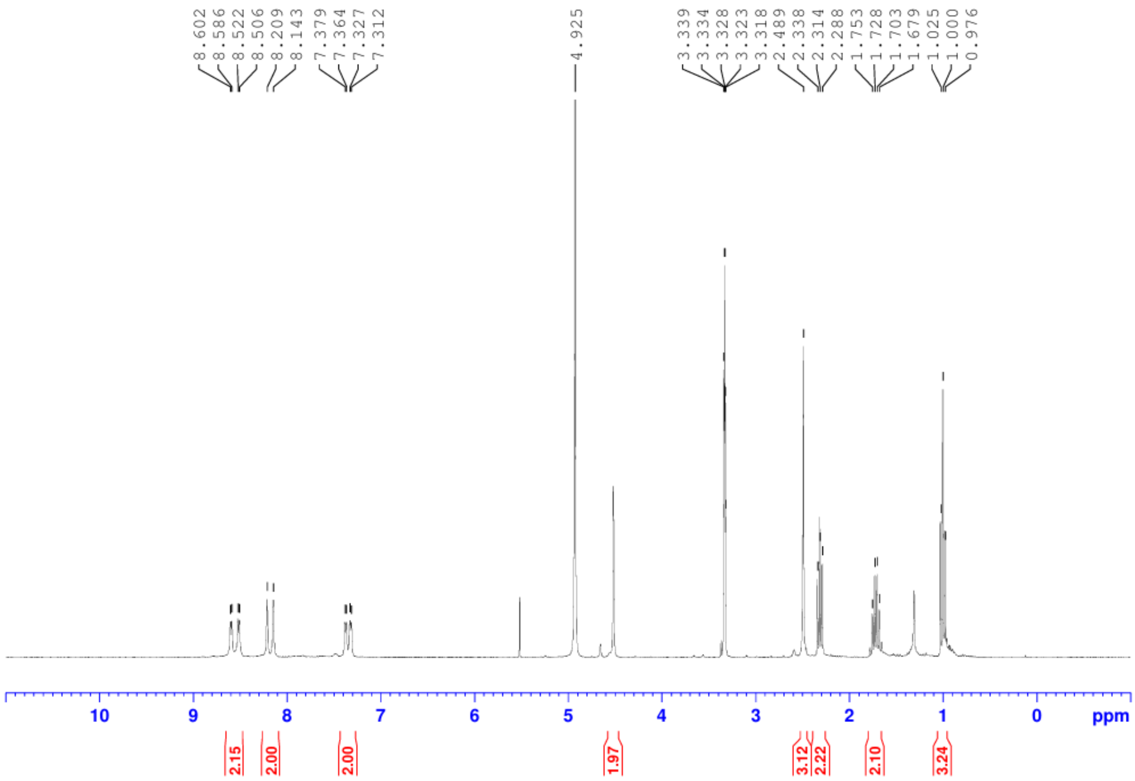

Figure S18. ^{1}H NMR spectrum of bpy-C4 in CD_3OD at 298 K.

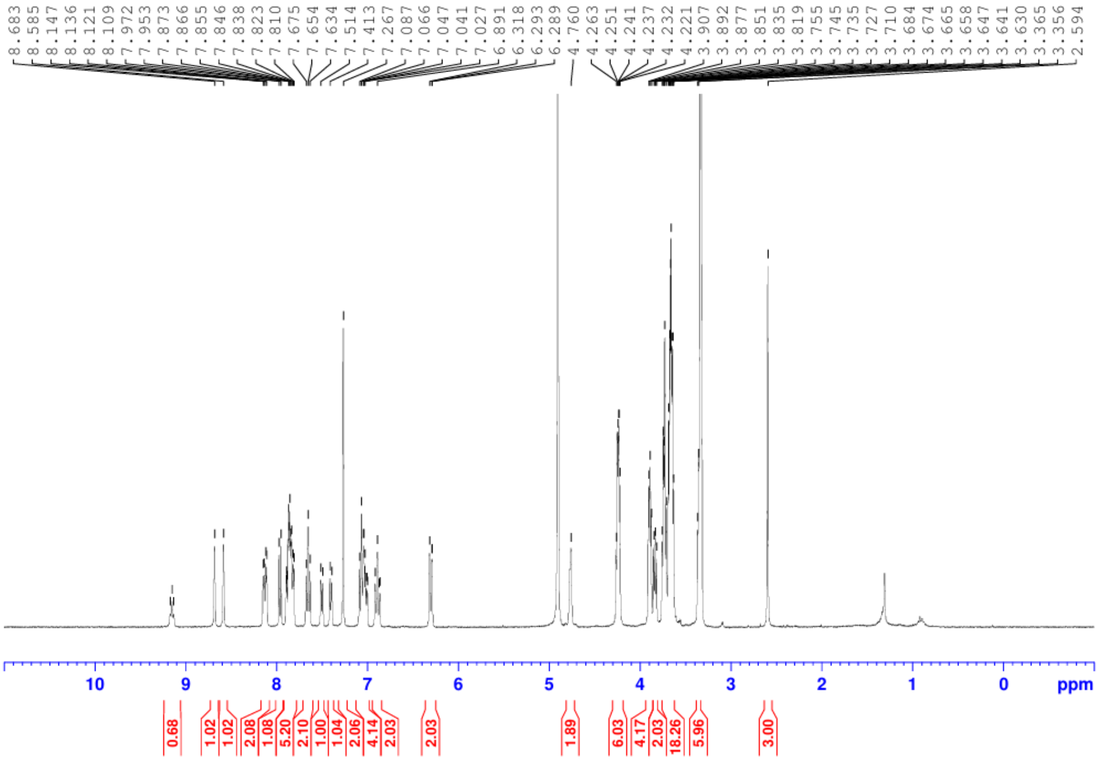

Figure S19. ^{1}H NMR spectrum of complex **1** in CD_3OD at 298 K.

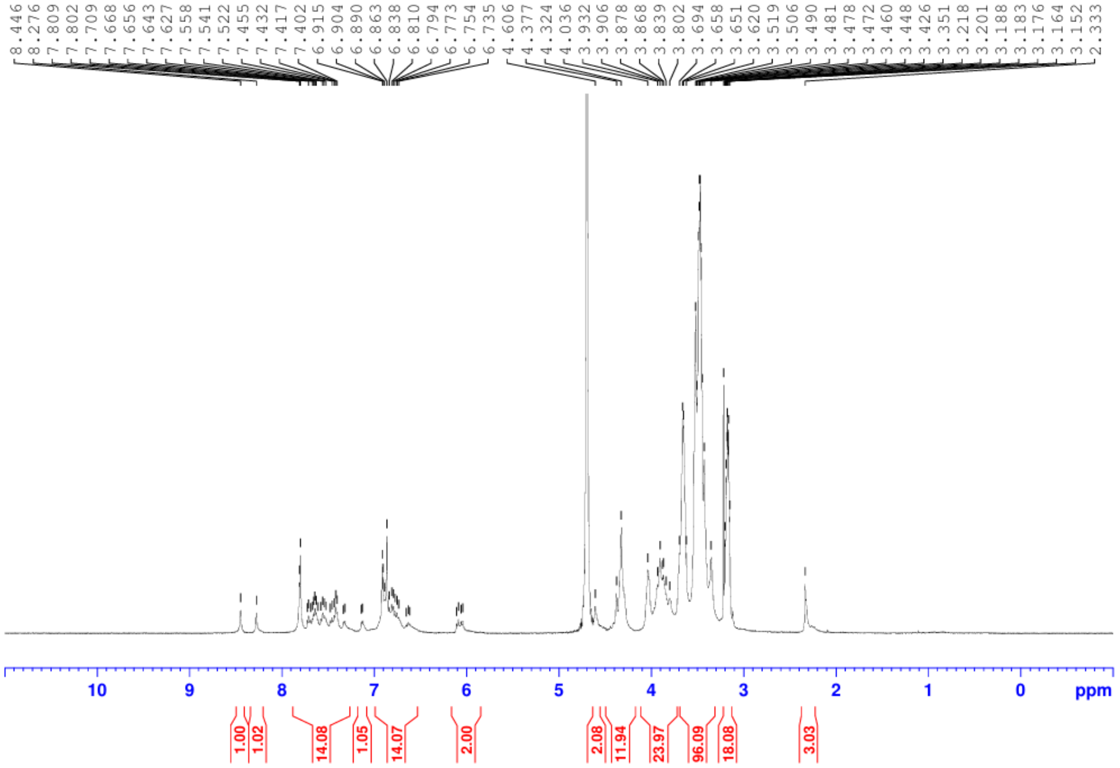

Figure S20. ^{1}H NMR spectrum of complex **1a** in D_2O at 298 K.

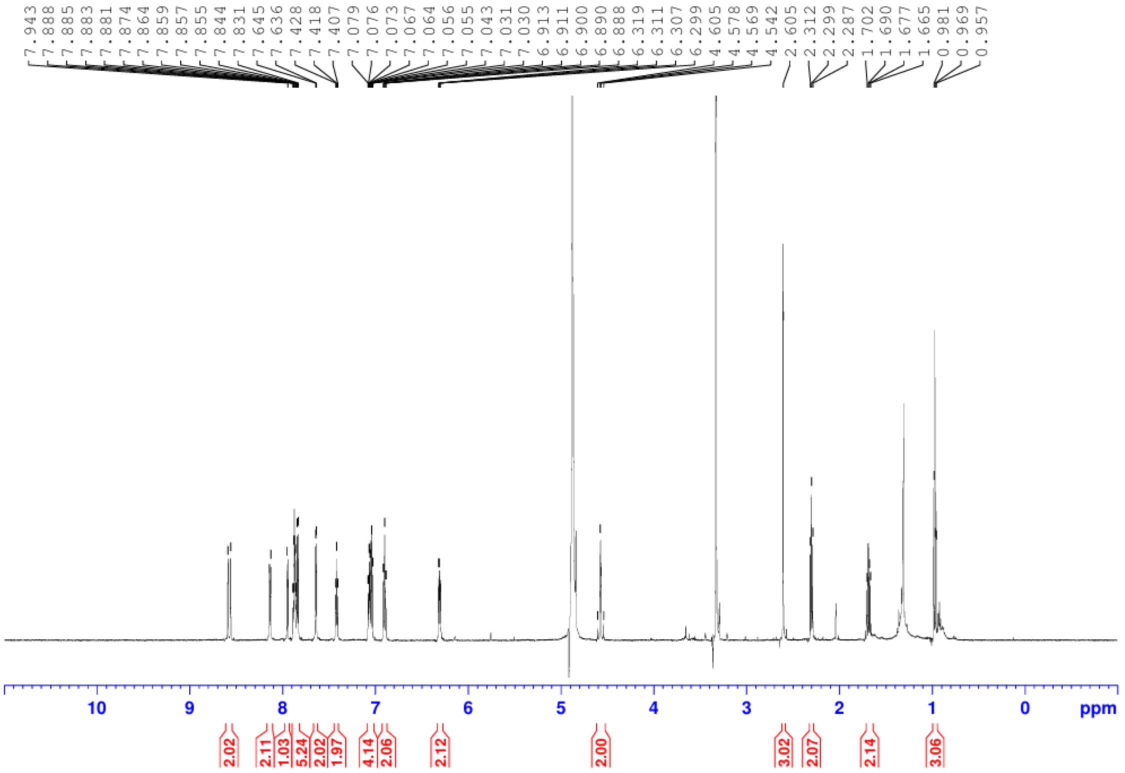

Figure S21. ^{1}H NMR spectrum of complex **1b** in CD_3OD at 298 K.

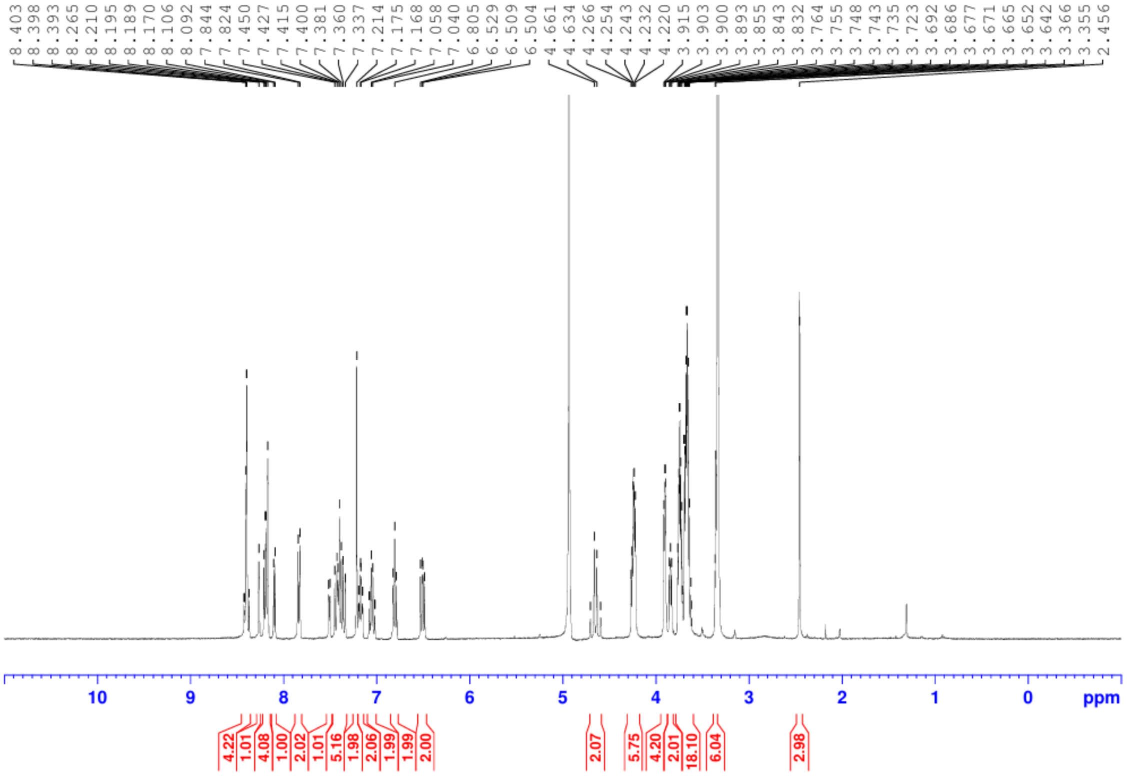

Figure S22. ^{1}H NMR spectrum of complex **2** in CD_3OD at 298 K.

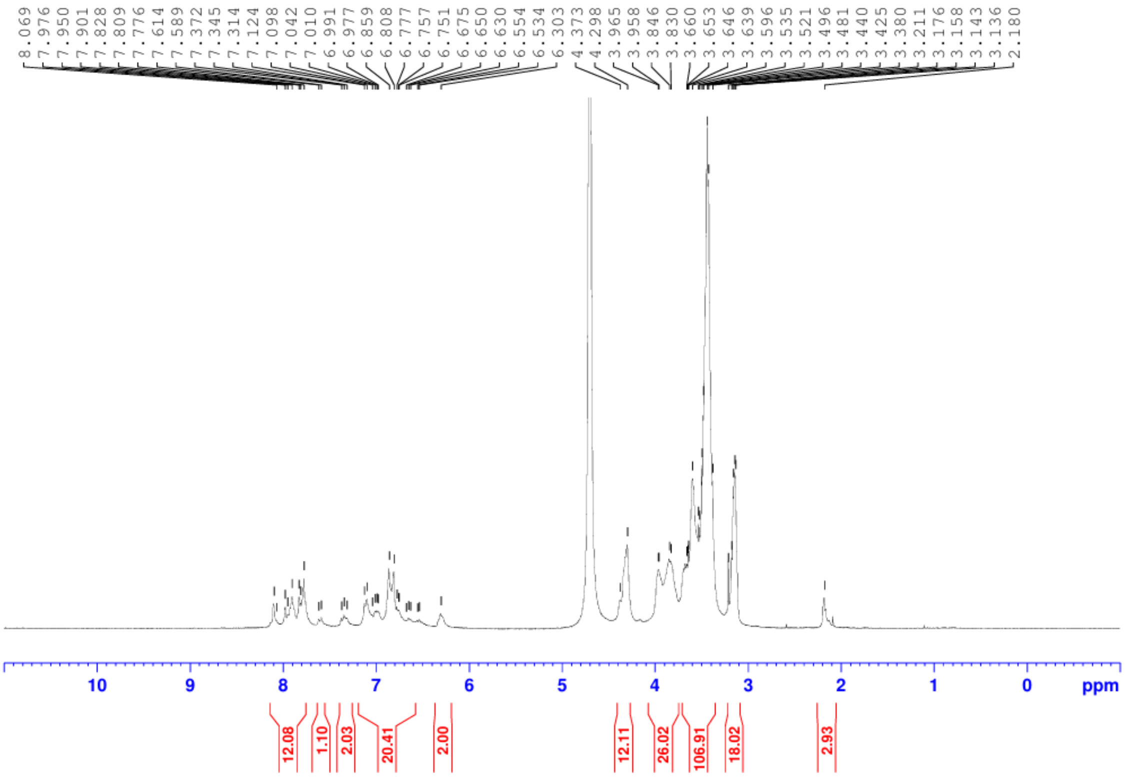

Figure S23. ^{1}H NMR spectrum of complex **2a** in D_2O at 298 K.

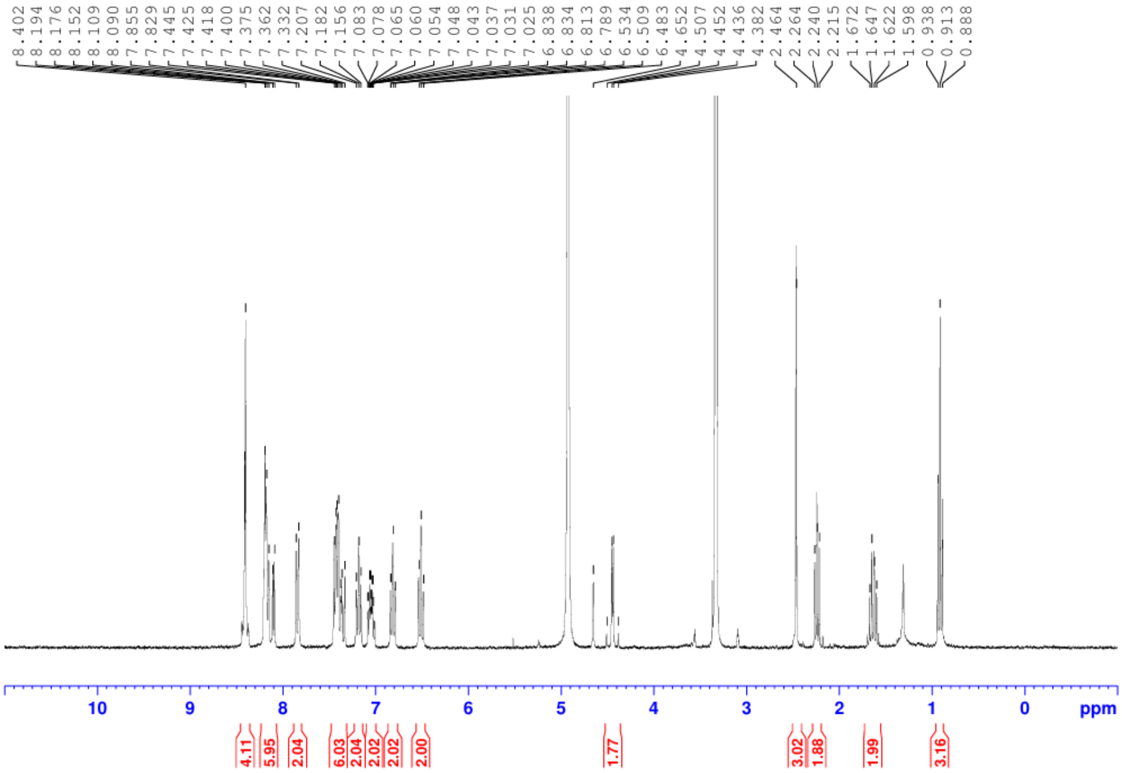

Figure S24. ^{1}H NMR spectrum of complex **2b** in CD_3OD at 298 K.

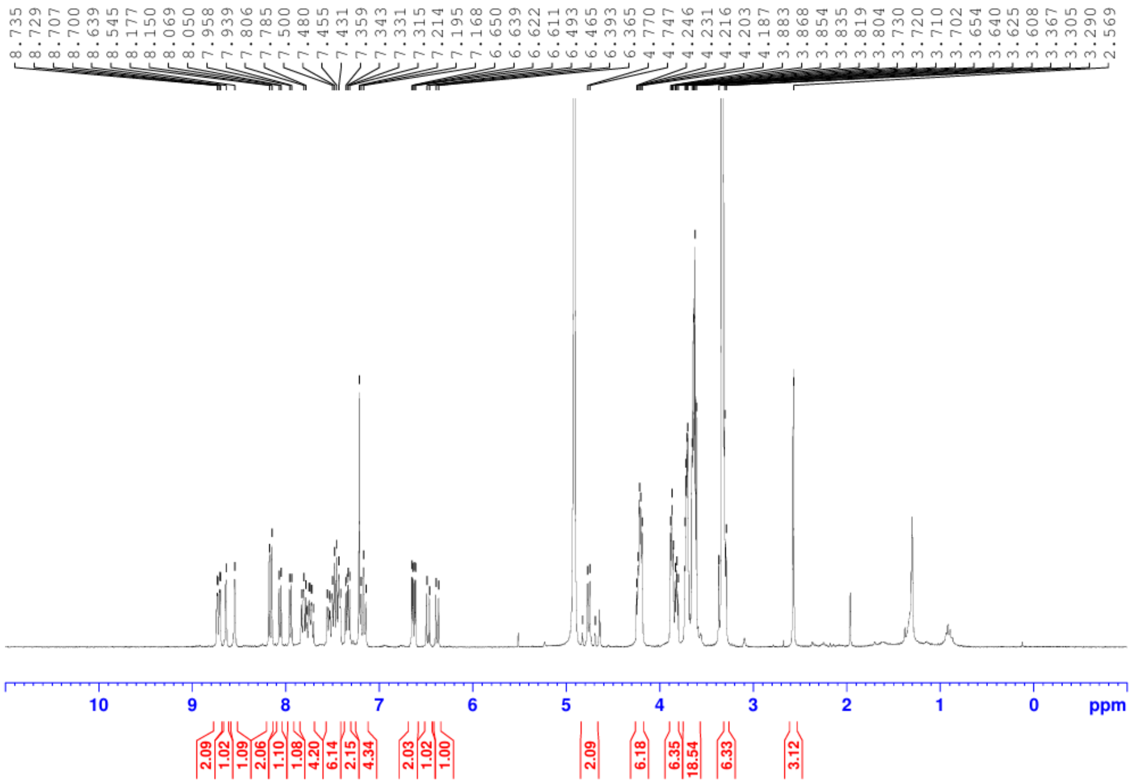

Figure S25. ^{1}H NMR spectrum of complex **3** in CD_3OD at 298 K.

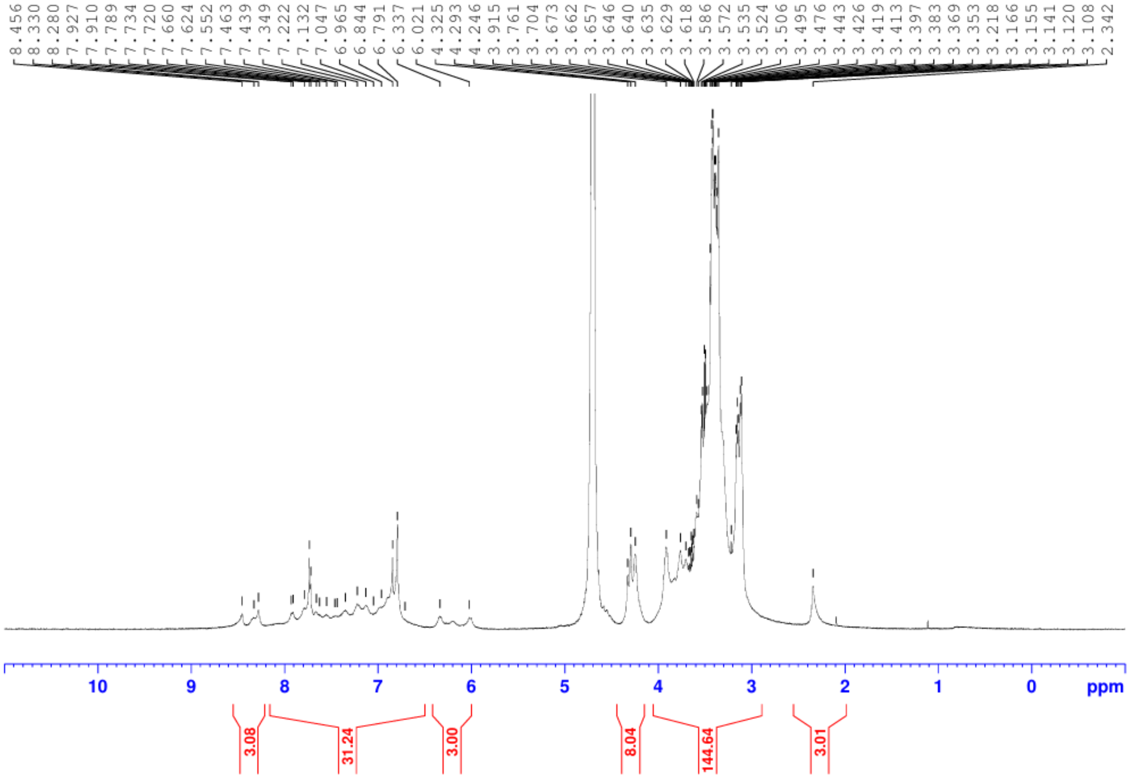

Figure S26. ^{1}H NMR spectrum of complex **3a** in D_2O at 298 K.

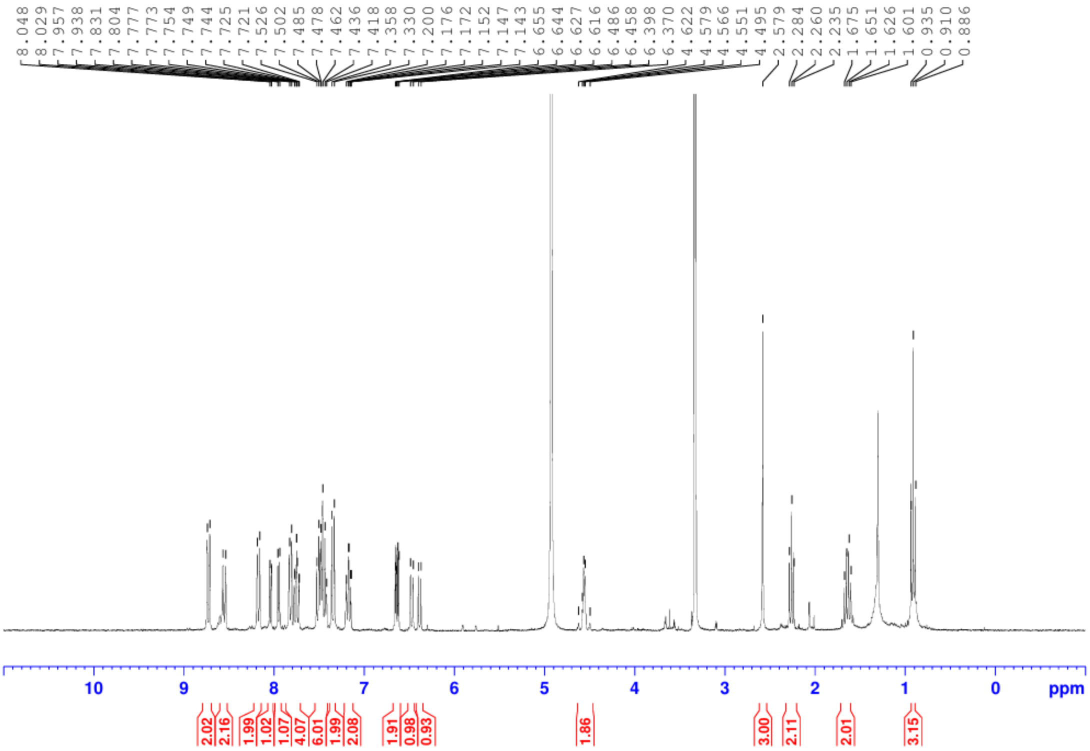

Figure S27. ^{1}H NMR spectrum of complex **3b** in CD_3OD at 298 K.

REFERENCES

(1) Meunier, S. J.; Wu, Q.; Wang, S.-N.; Roy, R. Synthesis of Hyperbranched Glycodendrimers Incorporating α-Thiosialosides Based on a Gallic Acid Core. *Can. J. Chem.* **1997**, *75*, 1472–1482.

(2) Busche, C.; Comba, P.; Mayboroda, A.; Wadepohl, H. Novel Ru^{II} Complexes with Bispidine-Based Bridging Ligands: Luminescence Sensing and Photocatalytic Properties. *Eur. J. Inorg. Chem.* **2010**, *2010*, 1295–1302.

(3) Suzuki, Y.; Okuro, K.; Takeuchi, T.; Aida, T. Friction-Mediated Dynamic Disordering of Phospholipid Membrane by Mechanical Motions of Photoresponsive Molecular Glue: Activation of Ion Permeation. *J. Am. Chem. Soc.* **2012**, *134*, 15273–15276.
